# Control of Fluoropolymer Crystallinity for Flexible, Transparent Optical Thin Films with Low Refractive Indexes

**DOI:** 10.1021/acs.macromol.4c02242

**Published:** 2025-01-29

**Authors:** Yineng Zhao, Fei Hu, Wyatt E. Tenhaeff

**Affiliations:** † Materials Science Program, University of Rochester, Rochester, New York 14627, United States; ‡ Department of Chemical Engineering, University of Rochester, Rochester, New York 14627, United States

## Abstract

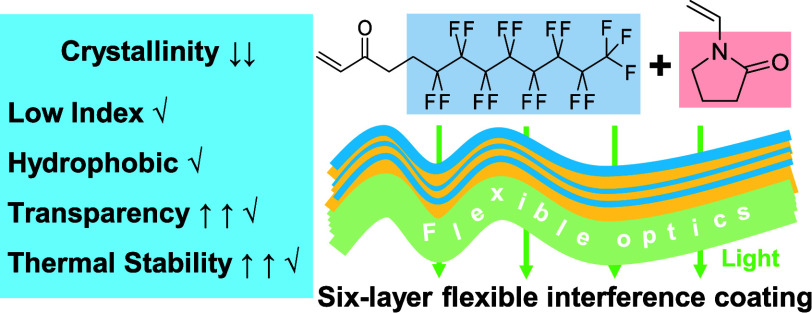

Fluoropolymers possess among the lowest indexes of refraction for dense, continuous materials, but their crystallinity typically leads to light scattering and haze. In this work, we studied poly(1*H*,1*H*,2*H*,2*H*-perfluorodecyl acrylate) (pPFDA) as a low-index fluoropolymer and successfully suppressed its crystallization while preserving its desirable low index of refraction (1.36 at 633 nm wavelength) and hydrophobicity (water contact angle of 122°). This was achieved through copolymerization between the hydrophobic 1*H*,1*H*,2*H*,2*H*-perfluorodecyl acrylate (PFDA) and *N*-vinylpyrrolidone (NVP) using initiated chemical vapor deposition (iCVD). The resulting copolymer p(PFDA-*co*-VP) film was smooth (roughness <2 nm), highly transparent, thermally robust, and mechanically flexible. This contrasted with pPFDA homopolymer films, which were rough (roughness >30 nm), hazy, and disintegrated at 70 °C due to melting. Moreover, the copolymerization resulted in a 16-fold improvement in the deposition kinetics. To demonstrate its excellent performance in practical applications, the low-index copolymer was paired with a high-index poly(divinylbenzene) (pDVB) (*n*_633_ = 1.59) to build a six-layer interference coating. A six-layer fully polymeric interference coating with precise, independent control of each individual layer’s thickness was prepared for the first time by iCVD. Optimized for broadband antireflection, it reduced the surface reflectance to 1% over the entire visible spectrum, while withstanding large mechanical strain.

## 1 Introduction

Low refractive index optical materials are critical components for next-generation, flexible, and responsive optical and optoelectronic devices, such as imaging systems, optical circuits, optical fibers, and waveguides.^1,2^ Low refractive index materials are typically fluorinated compounds due to the low atomic polarizability of fluorine.^3,4^ MgF_2_ has the lowest refractive index of 1.38 (*n* at 633 nm) among dense, continuous inorganic materials and is widely used in optical coating designs, such as antireflection layers.^1,5,6^ However, like other inorganic materials, MgF_2_ is brittle and will readily fracture in flexible devices.^7−9^ We have reported the fracture behavior of several conventional inorganic coatings (including MgF_2_, SiO_2_, and Al_2_O_3_) under biaxial strain on flexible, polymeric optical substrates.^7,10^ While ultralow indexes (<1.1, well below the index of MgF_2_) can be achieved in materials with high nanoscale porosity or by creating complex nanoscale patterns, the complex and costly fabrication methods and compromised physical robustness are significant limitations.^11−14^

Polymers, on the other hand, are mechanically flexible and compliant. As potential optical coatings, they possess elastic moduli and coefficients of thermal expansions that are comparable to their flexible optical substrates.^15^ Among polymers, fluoropolymers provide the lowest refractive indexes (mostly 1.3–1.4), even lower than that of MgF_2_.^3,4,16−18^ However, most fluoropolymers have compromised transparency due to a strong tendency for crystallization, which can lead to light scattering and haze.^16,19−21^ Polytetrafluoroethylene (PTFE or Teflon) and polyvinylidene difluoride (PVDF) have high fluorine contents and low refractive indexes but are also semicrystalline, rendering them opaque instead of transparent. This crystallization must be suppressed to successfully utilize fluoropolymers in optical applications. Many attempts have been made to suppress their crystallization through copolymerization, cross-linking, and many other strategies.^21,22^ The strategy employed in Teflon AF, wherein a cyclic comonomer (2,2-bistrifluoromethyl-4,5-difluoro-1,3-dioxole) is copolymerized with tetrafluoroethylene to disrupt the regular microstructure, is particularly effective in the suppression of crystallization and results in optically transparent films with a refractive index as low as 1.29.^17,23,24^ Despite the outstanding performance of Teflon AF, these backbone-fluorinated copolymers often require complex and proprietary manufacturing, making them extremely costly.^21,25^

Another important consideration in the development of optical coatings is processability. Highly fluorinated polymers, like PTFE and Teflon AF, are insoluble in most conventional solvents and require aggressive perfluorinated solvents, which complicates solution-based approaches to prepare optical coatings on polymeric optical substrates.^25,26^ Despite the capability of layer-by-layer assembly (LBL) in preparing polymeric multilayers with excellent thickness control of individual layers, its practical application has been restricted by the complex multistep solution processing and polyelectrolyte formulations.^27,28^ Sequential deposition of multiple optical coatings with independent nanometer-level thickness precision via solution approaches is another challenge.^29−35^ Backbone-fluorinated materials like Teflon AF can be fabricated into multilayers by vacuum deposition.^18,36,37^ However, the processes, such as physical vapor deposition (PVD) and laser ablation (LA), provide line-of-sight deposition; they do not conformally coat complex surface geometries.^38−42^ Also, they employ energetic sources such as plasma, heat, electron beams, or lasers to vaporize fluoropolymers that likely fragment sputtered polymers, leading to cross-linked coatings with ill-defined film compositions and chain microstructures. This is also a limitation of plasma-enhanced chemical vapor deposition (PECVD) of polymers.^43^ Finally, they can lead to significant temperature excursions in the polymer substrate, which result in residual stresses and severe mechanical distortion of the coating (e.g., wrinkles, buckles, etc.), and/or degradation of the temperature-sensitive substrate.^7,44−50^

Poly(perfluoro(meth)acrylates) are an alternative class of fluoropolymers with an aliphatic carbon backbone and perfluorinated pendant side chains. They possess low refractive indexes while having the advantage of facile synthesis due to the readily polymerizable (meth)acrylic-substituted vinyl groups.^51,52^ The synthesis of many fluoroacrylates has been demonstrated by initiated chemical vapor deposition (iCVD).^52−60^ iCVD synthesizes polymer thin films in situ with well-defined chemistries at near-ambient temperatures and has the ability to precisely control the thickness and create multilayer polymer stacks through sequential deposition. Hence, the technology is particularly well-suited for preparing polymeric optical coatings, as demonstrated in our previous studies.^7,52,60,61^ Among the fluoroacrylates, poly(1*H*,1*H*,2*H*,2*H*-perfluorodecyl acrylate) (pPFDA) has one of the highest atomic concentrations of fluorine, resulting in a reported refractive index of 1.36–1.37.^53^ However, the C8 fluorinated side chains of pPFDA can crystallize into hexagonal arrays, which consist of two layers of side chains vertically, resulting in a translucent film.^53,62,63^ The high crystallinity also gives pPFDA thin films a very rough surface as deposited, which may be a desirable feature when utilized as a hydrophobic coating but is an obstacle for optical applications.^63−67^ Moreover, the crystalline phase of pPFDA melts ca. 70 °C, which is not sufficiently high for further processing.^64,65,68,69^ If the film melts and then cools, the recrystallization can lead to film stresses and then dewetting and delamination of the films.

Cross-linking of pPFDA has been reported to suppress its crystallization.^64,66,70,71^ However, this strategy runs counter to the objective of low refractive indexes because it increases the film density and incorporates comonomers with higher polarizability (i.e., high molar refractivity).^64,66,72,73^ Despite the desirable combination of low refractive index, hydrophobicity, and ready preparation by iCVD, pPFDA has not been realized as a low refractive index optical coating. The major challenge lies in suppressing crystallization while maintaining a low index. In this study, PFDA was copolymerized with *N*-vinylpyrrolidone (NVP) to form copolymer thin films poly(1*H*,1*H*,2*H*,2*H*-perfluorodecyl acrylate-*co*-*N*-vinylpyrrolidone) via iCVD, abbreviated as p(PFDA*-co-*VP). Copolymers with different compositions were controlled by varying carrier gas flow ratios of NVP and PFDA during depositions and were subsequently labeled as pPFDA(*x*)-pVP(*y*) with *x* and *y* being the corresponding flow rates. The resulting films possess a refractive index as low as 1.365 at 633 nm and are highly transparent, homogeneous, smooth, and thermally stable due to its suppressed crystallization. Meanwhile, the hydrophobicity of pPFDA is also preserved for the copolymer with a water contact angle (WCA) larger than 120°, despite the drastically reduced roughness. The copolymer films withstood a prolonged annealing test with intact structural integrity and showed no sign of cracking or wrinkling. Finally, to validate the superior properties of the copolymer in a practical setup, a six-layer polymer multilayer was constructed on a 3 in. diameter polymeric lens with the thickness of each layer individually controlled to maximize a broadband antireflection effect over the entire visible range. A fully polymeric multilayer optical coating design is desirable for its excellent durability and flexibility.^28,74,75^ To achieve excellent optical performance, the individual layers must have tunable thicknesses, compositions, and optical properties, with excellent uniformity over large, practical areas. The six-layer coated device was then subjected to an aggressive strain test with the coating integrity and optical performance examined before and after the strain, showing excellent antireflection performance that reduced the surface reflectance to 1%.

## 2 Results and Discussions

### 2.1 Copolymerization by Initiated-Chemical Vapor Deposition (iCVD)

**Figure 1:**
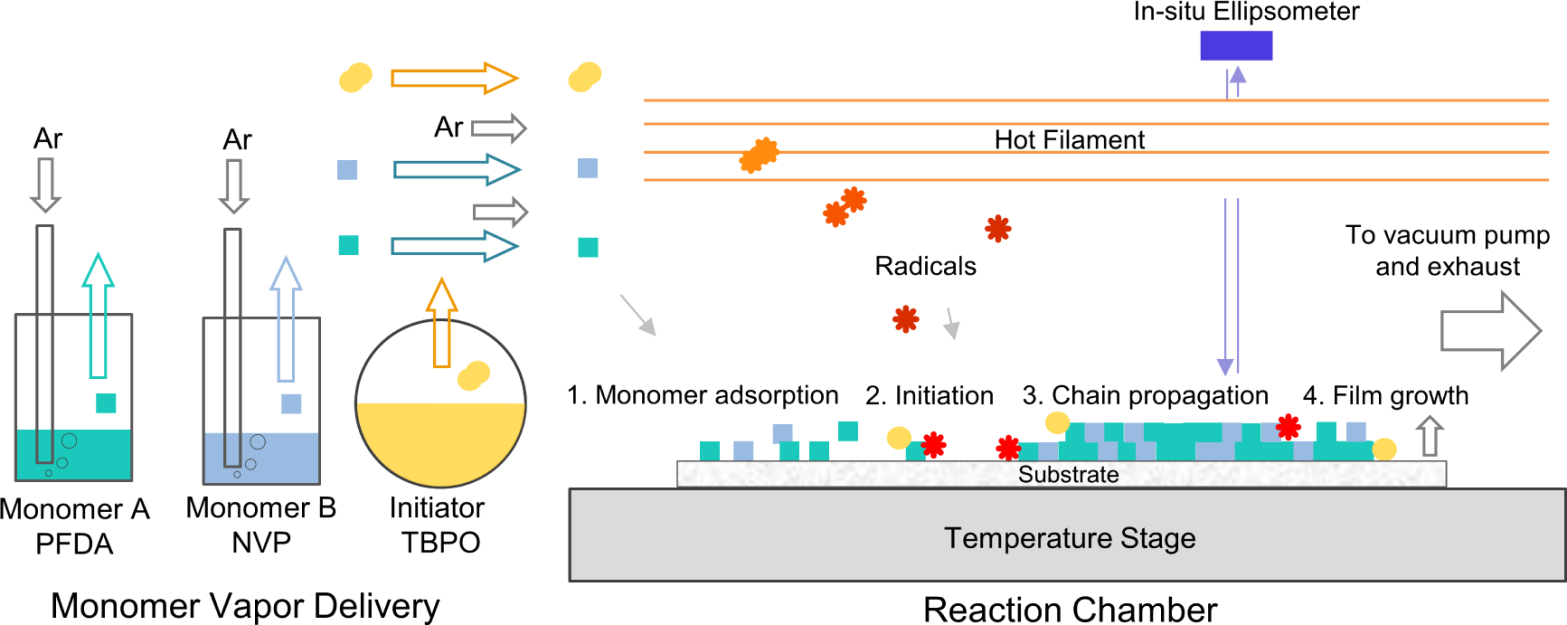
Cartoon illustration of film synthesis by iCVD and in situ monitoring of film thickness via spectroscopic ellipsometry.

**Figure 2:**
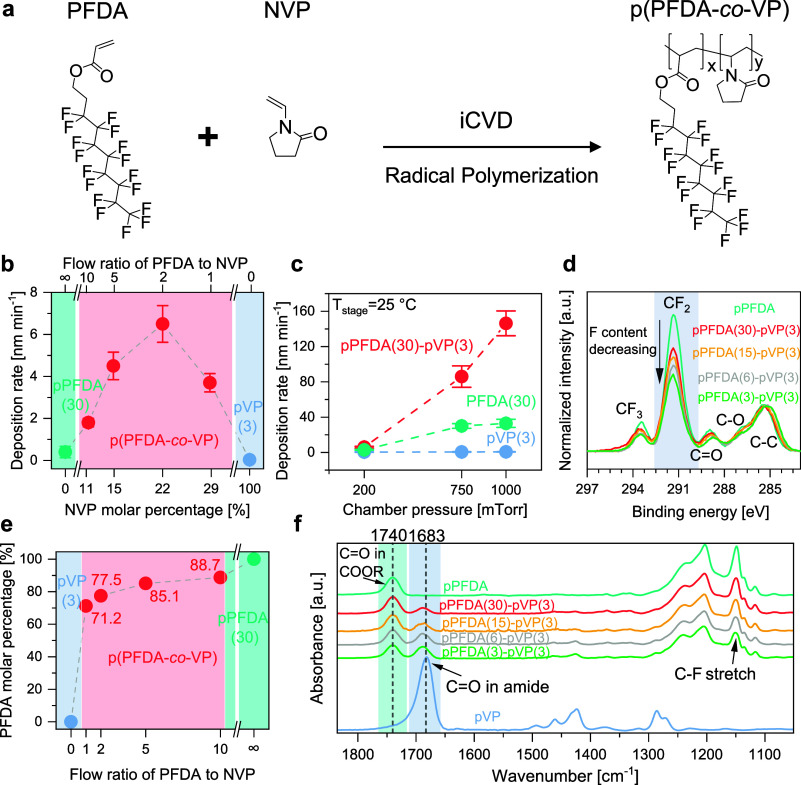
Kinetics and composition. (a) Reaction scheme of PFDA and NVP copolymerization forming p(PFDA*-co-*VP). (b) Comparison of deposition rates between homopolymerization and copolymerization as a function of NVP molar percentage in the deposited film. (c) Polymer film deposition rate vs chamber pressure. (d) XPS C 1s spectra show the trend of CF_2_ component peaks of the PFDA copolymer residues as a function of precursor flow ratios. (e) PFDA concentrations in the deposited films were calculated from the F content in the XPS survey results. (f) FTIR spectra of pPFDA, p(PFDA*-co-*VP), and pVP. The error bars in parts (b) and (c) represent standard deviation over triplicate measurements.

A conceptual depiction of the iCVD process is illustrated in Figure 1. Vapors of the 1*H*,1*H*,2*H*,2*H*-perfluorodecyl acrylate (PFDA) and *N*-vinylpyrrolidone (NVP) comonomers are independently and simultaneously delivered into a vacuum chamber via argon carrier gas. The ability to copolymerize these immiscible monomers, which lack an obvious common solvent, is a key processing advantage of iCVD. At the same time, the di-*tert*-butyl peroxide (TBPO) initiator is introduced into the vacuum chamber without the aid of carrier gas due to its high vapor pressure at room temperature and undergoes thermolysis at the Nichrome filament array suspended above the temperature-controlled stage.^76^ Physically adsorbed to the substrate surface, the monomers encounter initiator radicals, triggering heterogeneous radical copolymerization and film growth (Figure 2a). A spectroscopic ellipsometer integrated with the vacuum chamber enables in situ monitoring of film thickness and optical properties, providing precise control over each individual layer in a multilayered structure.

The depositions of pPFDA and pVP homopolymers by iCVD are well-studied.^22,53,62,77−80^ In this study, the kinetics of PFDA and NVP copolymerization were characterized not to optimize the deposition conditions for deposition rates or uniformity but to elucidate the heterogeneous copolymerization phenomena. In the first set of experiments, the chamber pressure was fixed at 200 mTorr and the stage temperature was set at 35 °C, while the carrier gas ratios of PFDA and NVP were adjusted to tune the molar concentrations of PFDA and NVP; the deposition rates are shown in Figure 2b. With the maximum flow rate of the PFDA carrier gas (30 sccm), the deposition rate of homopolymer pPFDA is only 0.4 nm min^–1^. The deposition rate of homopolymer pVP is even slower at 0.03 nm min^–1^—achieved with the maximum flow rate of the NVP carrier gas (3.0 sccm). The low deposition rate can be partially attributed to a low filament current and chamber pressure, which were used to ensure the deposition of smooth films amenable to characterization by ellipsometry.^62,65^ By introducing the comonomers together (30 sccm of PFDA, 3 sccm of NVP), the deposition rate increased to 1.8 nm min^–1^. Further reducing the flow rate of PFDA to 6 sccm results in a deposition rate as high as 6.5 nm min^–1^. The copolymerization kinetics at higher chamber pressures and lower stage temperatures were also investigated, as these conditions may enhance deposition rates. A higher pressure may improve the bubbler efficiency, hence increasing monomer vapor pressure (*p*_m_) in the chamber, and a reduced substrate temperature decreases the saturation pressure of the monomer at the surface (*p*_sat_). As a result, the ratio of *p*_m_/*p*_sat_ increases, which results in higher iCVD deposition rates in most scenarios. The deposition rates under these conditions are reported in Figure 2c. Increasing the chamber pressure to 1000 mTorr yields a deposition rate of 33 nm min^–1^ for the pPFDA homopolymer and 0.65 nm min^–1^ for the pVP homopolymer. When copolymerizing PFDA (30 sccm) and NVP (3 sccm) at 1000 mTorr, the deposition kinetics reached 146 nm min^–1^. The superior deposition rate of the copolymerization at comparable deposition conditions may suggest a higher crossover rate constant in the copolymerization. Because the efficiencies of the bubblers in the iCVD system are not 100% and can vary significantly with processing conditions, it is difficult to reliably estimate the partial pressures of the comonomers during the deposition. Hence, the relative surface concentrations of the comonomers are unknown, and the exact relationship of the rate constants cannot be determined. However, general expectations can be inferred from the *Q*–*e* scheme, wherein copolymerization reactivity ratios are calculated using tabulated reactivity and polarity values for comonomers. NVP has an *e* value of −1.62 (*Q* = 0.088), indicating an electron abundance to the vinyl C=C double bond caused by the electron-donating side group.^81^ Because the *e* value of PFDA is not readily available, 1*H*,1*H*-heptafluorobutyl acrylate with a shorter fluorinated side chain served as a proxy. The estimated *e* for PFDA is +1.34 (*Q* = 0.96), suggesting an electron-deficient double bond of PFDA with its longer and stronger electron-withdrawing pendant group.^81^ The calculated reactivity ratio *r*_1_ of the fluoroacrylate homopolymerization over copolymerizing with NVP is estimated to be *r*_1_ = *k*_11_/*k*_12_ = 0.21 while the corresponding *r*_2_ = *k*_22_/*k*_21_ = 0.00076. The details of the calculation are included in the Supporting Information. Clearly, crossover propagation is favored especially for NVP.^82^ The fact that *r*_1_ is smaller than 1 indicates the fluoromonomer has a faster rate constant for copolymerizing with NVP than homopolymerization. This may be the explanation for the faster deposition rate after introducing NVP. Alternatively, a complex may be formed by the electronic interaction between the electron-accepting PFDA and the electron-donating NVP.^83^ This enhancement in copolymerization kinetics has been observed in previous iCVD studies.^84−86^

The copolymer film composition was characterized by XPS and FTIR. In the F 1s high-resolution spectrum (Figure 2d), the relative intensity of the component peak assigned to CF_2_, associated with the perfluorinated side chain of PFDA, decreases with the flow rate of PFDA. The reduced PFDA flow rates translate to reduced PFDA surface concentrations and less incorporation into the copolymer chain. The quantitative copolymer compositions derived from the XPS survey scans (Figure S1) are shown in Figure 2e. Using comparable carrier gas flow rates of PFDA and NVP, the PFDA constitutes 71 mol % of the copolymer composition; it approaches 90 mol % when the flow ratio of PFDA to NVP carrier gases is 10 (30–3 sccm). The preferential incorporation of the PFDA comonomer, which is desirable for achieving a high fluorine content for low refractive indexes and surface energy (hydrophobicity), is a consequence of the superior homopolymerization kinetics of fluoroacrylate over NVP—revealed by *r*_1_ being 3 orders of magnitude larger than *r*_2_ (though these values are estimates).

FTIR spectra (Figure 2f) of the homopolymer and copolymers were analyzed to further understand the chemical bonding within the materials. The peak intensities are normalized to film thickness to facilitate quantitative FTIR analysis. Several characteristic peaks can be observed for pPFDA, such as C=O stretching from ester groups at 1740 cm^–1^ and a group of C–F stretches at 1235, 1203, and 1149 cm^–1^.^22,87^ PVP shows a strong peak at 1683 cm^–1^, which is attributed to the stretching mode of C=O in the cyclic amide.^79^ The C=O peaks at 1740 and 1683 cm^–1^ are characteristic of pPFDA and PVP, respectively, and do not overlap with any peaks of their respective counterparts, which makes these two peaks a convenient indicator of copolymer composition. Upon copolymerization of PFDA and NVP, the intensity of the C=O mode from the ester group in PFDA only slightly reduced, with an addition of an amide C=O peak next to it, consistent with the trend of dominating PFDA concentration in copolymer by XPS. According to the Beer–Lambert law, the absorbance is proportional to the molar concentration of a constituent. The C=O (1740 cm^–1^) peak area in homo-pPFDA represents 100% PFDA molar concentration, so PFDA concentration in copolymers can be identified by the area ratio of C=O from the PFDA moiety in a copolymer to that in the homo-pPFDA through quantitative FTIR. pPFDA(3)-pVP(3), pPFDA(6)-pVP(3), pPFDA(15)-pVP(3), and pPFDA(30)-pVP(3) revealed a PFDA concentration of 66.5%, 79.0%, 84.1%, and 91.7%, respectively, which is consistent with the PFDA concentration quantified by XPS. All other peaks of homo-pPFDA and homo-pVP are present in the copolymer spectra, with their intensity following the expected trend with the concentrations. Such results from FTIR not only confirmed the chemical structure of iCVD-created copolymer p(PFDA*-co-*VP) but also corroborated the compositions measured by XPS.

### 2.2 Crystallization and Thermal Properties

**Figure 3:**
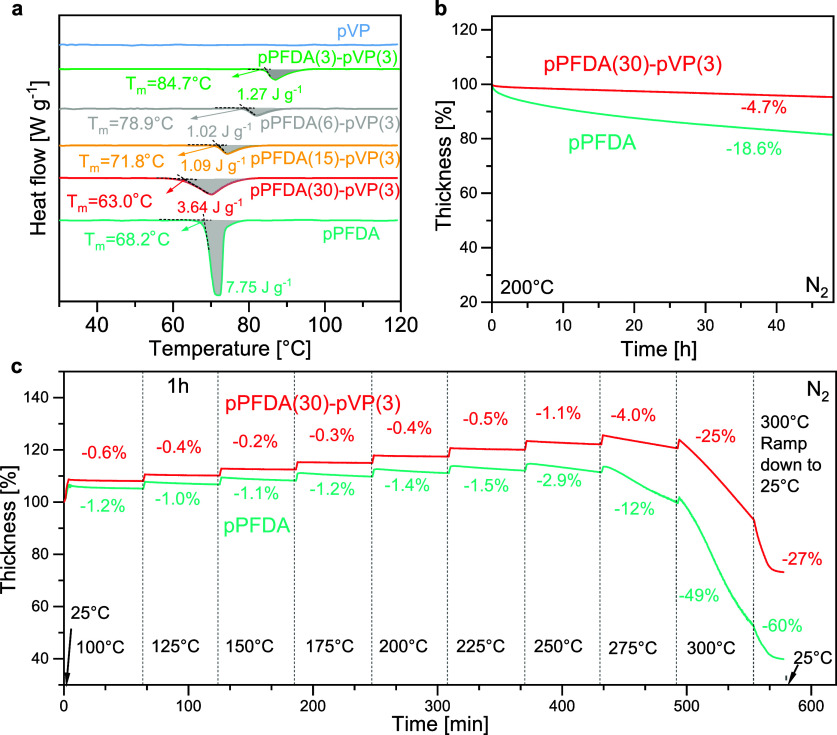
Thermal properties. (a) DSC heat flow curves of the first heating of the pPFDA homopolymer and p(PFDA*-co-*VP) copolymer samples. The DSC ramp rate is 5 °C min^–1^ in a N_2_ atmosphere. Thickness evolution of polymer films on Si at (b) a constant temperature of 200 °C and (c) stepwise isothermal conditions, monitored by in situ ellipsometry. The ramp rate between set isothermal temperatures is 20 °C min^–1^ in a N_2_ atmosphere. All samples had nominal film thicknesses of 100 nm, and normalized thicknesses were reported in the figures to aid comparison.

The primary objective for incorporating NVP into pPFDA through copolymerization was to suppress the crystallization of the PFDA moiety. To evaluate the success of this strategy, differential scanning calorimetry (DSC) was used to study the crystallization behavior of pPFDA and the copolymers. Samples were scratched from the Si wafer substrate and measured without additional heat treatment to preserve the crystallographic information of the as-deposited thin films. The heat flow curves of the first heating were shown in Figure 3a with normalized baselines.^88−90^ An endothermic peak associated with the melting of the crystalline phase of perfluorodecyl pendant groups was observed for the pPFDA homopolymer and all the copolymers.^91^ In contrast, the heat-flow curve of homo-PVP was a featureless straight line, indicating no phase or glass transition in this temperature window. The glass transition temperature (*T*_g_) of pVP is usually above 150 °C.^92^ The pPFDA homopolymer reveals a sharp melting endotherm starting at 68.2 °C with an enthalpy of fusion of 7.75 J g^–1^, as anticipated for the crystalline behavior of pPFDA. With increasing incorporation of NVP, the enthalpy of fusion was greatly reduced, as demonstrated by the shrinking endothermic peaks. Meanwhile, a shift of peak position or melting temperature was also observed. The result is also summarized in Figure S2a with reference to the NVP concentration in the copolymers. For NVP contents of 11%, 15%, and 22 mol % in the copolymer, the enthalpy of fusion was reduced to 3.64, 1.09, and 1.02 J g^–1^, respectively. Conventionally, the degree of crystallinity of a semicrystalline polymer can be calculated from the fraction of the DSC-measured enthalpy of fusion over the theoretical enthalpy of fusion of the “ideal” 100% crystalline phase of that polymer.^93^ As such, the crystallinity is proportional to the enthalpy of fusion. Due to the lack of reference data on the fusion enthalpy of a pure pPFDA crystal, the absolute crystallinity value cannot be determined. However, the relative reduction of crystallinity can be calculated for copolymers based on the fractional reduction of their enthalpy of fusion. For the copolymers with NVP molar percentages of 11%, 15%, and 22%, the crystallinity was reduced to 47%, 14%, and 13% of that of homo-pPFDA. Increasing the NVP content above 15 mol% only slight reducted the fusion enthalpy. X-ray diffraction (XRD) (Figure S3) was measured as well, and the crystallinity reduction of the copolymer relative to pPFDA can be approximately measured by calculating peak area differences at 5.4° (2θ). The copolymers with NVP content (mol %) of 11%, 15%, 22%, and 29% had 61.4%, 13.8%, 9.7%, and 5.7% crystallinity relative to that of the homo-pPFDA, respectively. Both XRD and DSC showed the same success in crystallization suppression by copolymerization, and the values of crystallinity reduction were in general agreement with each other. Crystallinity was largely suppressed with 15 mol% NVP inclusion; more NVP produced diminishing results, and only a slight amount of crystalline phase remained.

Most semicrystalline polymers have a crystallinity lower than 80%.^94^ In 2012, Coclite et al. reported the crystallinity index of iCVD-pPFDA ranging from 15% to 75% as a function of deposition conditions.^62^ Using this as a reference and assuming the crystallinity of homo-pPFDA was 75% as a conservative guess, the degree of crystallinity of the copolymer with 15 mol % NVP or more would have a crystallinity of less than 10%. This result indicated that copolymerizing PFDA with NVP is a successful and efficient strategy for crystallization suppression of PFDA. Keeping the NVP at a low concentration is beneficial to maintaining a high fluorine concentration to preserve the desired properties of a low refractive index and hydrophobicity. The onset temperature of crystalline phase melting was determined to be 68.2 °C for homo-pPFDA by iCVD. After incorporating NVP of 11%, 15%, 22%, and 29% mol, the melting temperature first reduces to 63.0 °C and then increases to 71.8, 78.9, and 84.7 °C. The melting-point depression after incorporating 11% NVP is consistent with the expectation from copolymerization. However, further incorporation of NVP increased the melting temperature. This may result from the increased backbone rigidity and polarity of the NVP. The higher melting temperature comes from a very small amount of crystalline phase (less than 10%) that was not disrupted by the presence of NVP, so it may not be representative of the bulk behavior of the polymer, which is dominated by the amorphous phase instead of the residual crystalline phase.

To assess the thermal stability, pPFDA(30)-pVP(3) and homo-pPFDA were kept isothermal at 200 °C for 2 days (48 h), and their thickness evolution was recorded in Figure 3b. The pPFDA homopolymer lost over 18.6% of its thickness while pPFDA(30)-pVP(3) only reduced by 4.9%.. To further study the thermal stability under elevated temperatures, a stepwise heating ramp test was performed on a polymer thin film deposited on a Si wafer, and the thickness evolution was compared. pPFDA(30)-pVP(3) was chosen as the representative copolymer in this thermal test for its smallest NVP concentration which may imply the smallest property change due to the NVP incorporation. As shown in Figure 3c, pPFDA and pPFDA(30)-pVP(3) with the same thickness of 100 nm were ramped up to 100 °C at a rate of 20 °C min^–1^ and held for 1 h. Both exhibited large thickness increments after heating due to typical thermal expansion. After the 1 h soak, negligible thickness change (−0.4%) was observed for the copolymer, pPFDA(30)-pVP(3), but the pPFDA homopolymer revealed a larger thickness decrease of −1.2%. The temperature then increased stepwise from 100 to 300 °C with intervals of 25 °C with 1 h soaks. The copolymer showed a smaller thickness reduction than the homopolymer at every temperature. Starting from 275 °C, thermal decomposition became obvious for both, but the homopolymer showed a drastic thickness loss of 12% compared to that of only 4% for the copolymer, and the homo-pPFDA lost half of its thickness at 300 °C while the copolymer lost a quarter. After cooling from 300 to 25 °C, the cumulative thickness reduction in the copolymer was 29%, compared to 60% in the homopolymer. With the inclusion of 15 mol % NVP, pPFDA(15)-pVP(3) showed almost identical behavior as pPFDA(30)-pVP(3) in this test, as shown in Figure S2b. It is evident that the introduction of NVP as a copolymer component to PFDA even in small quantities not only suppressed the crystallization but also greatly improved the thermal stability and enabled it to survive longer time at higher temperatures. Such improvement could result from a possible higher molecular weight, which was implied by the better kinetics of the copolymerization compared with their respective homopolymerization. However, measuring the molecular weight characterization of the copolymer is beyond the scope of this report, and the mechanisms of thermal degradation in these materials will be the subject of future studies.

### 2.3 Surface Properties

**Figure 4:**
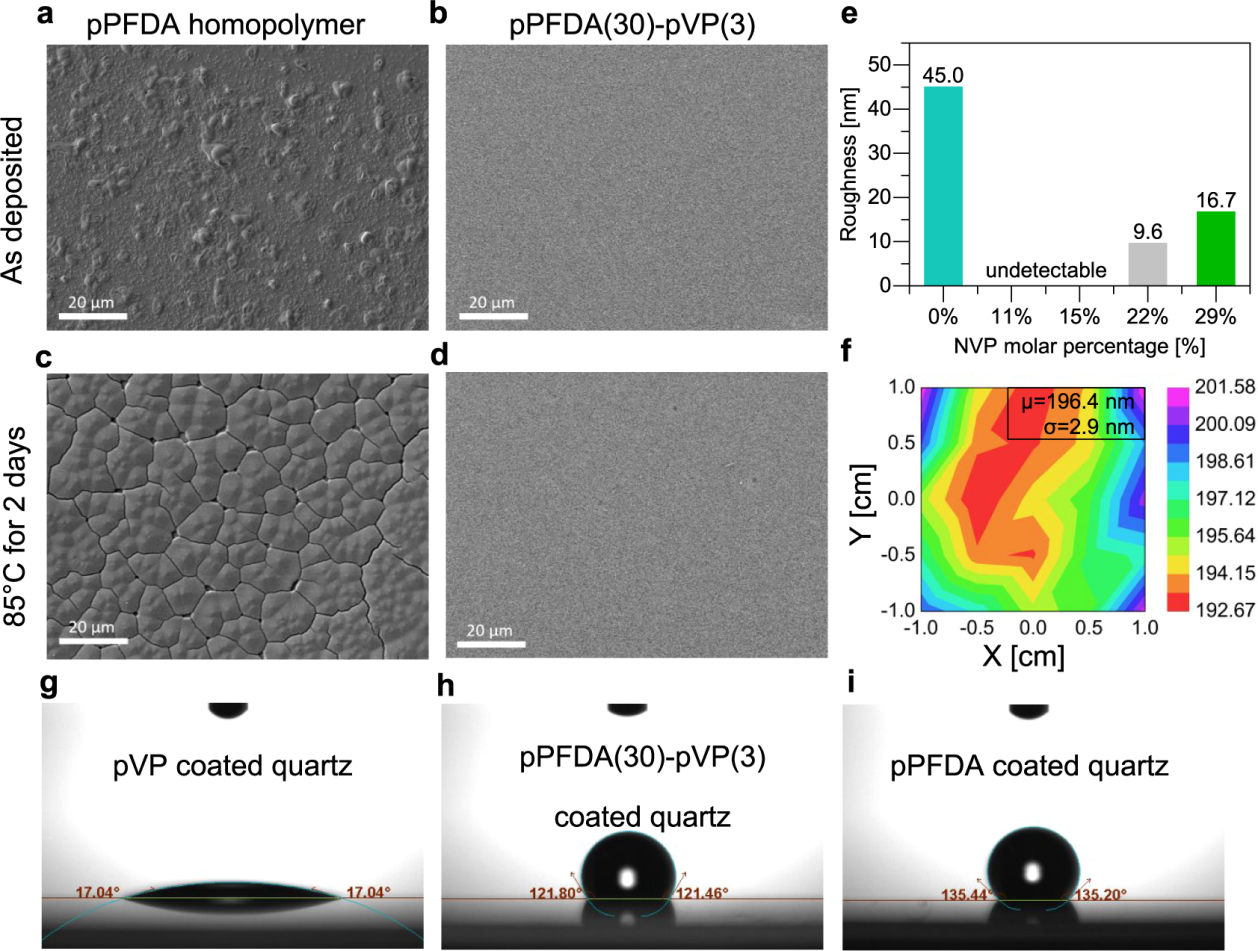
Surface properties. SEM images of (a, c) pPFDA homopolymer and (b, d) pPFDA(30)-pVP(3) copolymer (a, b) before and (c, d) after annealing at 85 °C for 2 days. (e) Roughness of 100 nm film on Si made by pPFDA and p(PFDA-*co*-VP) copolymers at different compositions. (f) Thickness mapping of pPFDA(30)-pVP(3) film on quartz. Water contact angles of (g) the pVP film, (h) pPFDA(30)-pVP(3), and (i) pPFDA films deposited on quartz.

An ideal optical thin film should have a smooth surface topography and little to no bulk heterogeneities that scatter light. To minimize Mie scattering, any heterogeneities (defects, second phases, pores, etc.) should be smaller than l/10 ∼ l/20 of the incident wavelengths (20–40 nm for visible light). Scanning electron microscopy (SEM) of homo-pPFDA revealed a rough, granular surface morphology (Figure 4a). The granular morphologies can be characterized by two length scales—smaller particles about 100 nm in size and large blisters around 2–3 μm. In both cases, these features are large enough to scatter light, and the films appear translucent when coated on transparent substrates. The feature is suspected to be small pPFDA crystallites, whereas the blisters form through the aggregation of the crystallites. Given the room temperature deposition of pPFDA (below its melting point), the deposited films likely do not possess enough mobility to sustain extended crystal growth. The film was imaged again at room temperature (Figure 4c) after annealing at 85 °C (above its melting temperature at 69 °C) for 2 days. The film is clearly fractured into many small segments. Each segment is about 2 μm in diameter and has a pointed center, with straight marks radiating to its edge. The same features were previously reported by Lacroix-Desmazes et al., suggesting the presence of spherulitic crystal morphologies.^95^

Macroscopic dewetting of the annealed film also occurred upon annealing, leading to large areas of exposed substrate (Figures S4 and S5). Polarized optical microscopy at the edges of the retreating films (Figure S6) revealed Maltese cross patterns that are characteristic of spherulites, further supporting their existence in annealed pPFDA. The cracks between crystallites observed in SEM and pinholes were formed at some boundaries. Upon annealing, the film underwent recrystallization, which generated large internal stress by producing crystallites at a larger density, resulting in the densification and shrinkage of the annealed film. This also led to film defects, such as the holes and cracks observed in Figures S4–S6. As a result, the crystallization of pPFDA not only interfered with light transmission but also caused the entire polymer film to lose structural integrity and disintegrate when annealed above its melting temperature. This essentially rendered the film unusable and significantly compromised the processability and working temperature range.

In contrast, the copolymer pPFDA(30)-pVP(3) with 11 mol % NVP inclusion possessed a featureless and smooth surface before (Figure 4b) and after annealing (Figure 4d). No crystallites, cracks, pinholes, or other inherent defects were observed by SEM. Despite the copolymers possessing some crystallinity, it was sufficiently suppressed such that the crystallites likely remained small and the film did not undergo significant thermal stress during annealing. The POM images of other copolymers were also displayed in Figure S7, which showed smooth surfaces contrary to pPFDA. This result is highly favorable for the potential application of these materials as optical coatings as the melting point no longer limits the processing temperatures. The upper limit of the working temperature of the copolymer can be defined by its thermal decomposition temperature (250 °C).

Ellipsometry was also employed to quantitatively compare the roughness and thickness uniformity of films deposited on the quartz substrates. Figure 4e compares the roughness of as-deposited 100 nm films as a function of NVP concentration. The pPFDA homopolymer reveals the highest surface roughness of 45.0 nm due to its high crystallinity, while insignificant roughness was observed for two copolymer species containing 11% and 15% NVP. As the NVP molar concentration increased to 22% and 29%, the roughness of the as-deposited films increased to 9.6 and 16.7 nm, respectively. SEM of pPFDA(6)-pVP(3) (Figure S8a), possessing 22 mol % NVP, revealed a layer of particulate structures deposited on the film surface. This morphology is unlikely due to crystallization, since both DSC and XRD confirmed that crystallinity is heavily suppressed in these compositions. Rather, the rough surface morphology of the copolymer films with the highest VP content is a complex function of processing parameters and film composition. Previous studies have shown that pPFDA film morphology is dependent on deposition kinetics (e.g., monomer surface concentrations, filament temperatures, and initiator concentrations), extent of crystallinity, cross-linking, and copolymer compositions.^62,64,66,96^ Interestingly, iCVD-deposited films of 1*H*,1*H*,2*H*,2*H*-perfluorooctyl acrylate (pPFOA) homopolymers are amorphous and smooth but become rough when cross-linked with divinylbenzene, suggesting that monomer aggregation/separation on the surface during the deposition may contribute to roughness.^56^ The poly(divinylbenzene) films display negligible roughness. In the copolymers of p(PFDA-*co*-VP) prepared in this study, both monomer aggregation and the deposition kinetics, which have a complex dependence on flow ratios (Figure 2e), likely influence surface morphology. Surprisingly, however, the rough copolymer films are smoothed by thermal annealing (Figure S8b); the particulates on the surface disappear. This is enabled by the linear backbone of the copolymers and contrasts with previous studies on semicrystalline or cross-linked PFDA, wherein thermal annealing can reduce roughness to a degree but does not fully eliminate it.^62,64,66^ The mechanisms for this roughness development and subsequent smoothing with thermal annealing are beyond the scope of the current study. A thickness nonuniformity (Figure 4f) of only 2.9 nm was measured in a pPFDA(30)-pVP(3) film deposited over a 2 × 2 cm area on quartz, with an average thickness of 196.4 nm. This indicates that the copolymer film deposited by iCVD can achieve great thickness uniformity over a meaningful area relevant to optical devices.

Hydrophobicity is another desired property of optical coatings, as it reduces scattering from water droplets or fogging. pPFDA has long been studied as a hydrophobic coating material due to the long perfluorinated side chain.^97^ Water contact angles (WCA) were measured to characterize the effect of NVP on the surface energies of the films. Bare quartz without any polymer coating had a WCA of ∼58° (Figure S9)—somewhat hydrophilic. PVP, on the other hand, formed a highly hydrophilic surface with an extremely low water contact angle of ∼17° (Figure 4g) owing to its polar pyrrolidone group. In fact, the water droplet was completely absorbed into the PVP film within seconds of contact. Consistent with previous reports, pPFDA films made the surface hydrophobic with a WCA of ∼135° (Figure 4i), slightly larger than the 120° reported in the literature, likely due to its rough surface.^97^ Rougher surfaces usually offer better hydrophobicity by trapping more air but are highly undesirable for optical coatings due to surface haze (light scattering).^10,98^ After copolymerizing with NVP, the copolymer, pPFDA(30)-pVP(3), maintained the outstanding hydrophobicity from pPFDA and showed a WCA of 121° (Figure 4h) despite the incorporation of extremely hydrophilic PVP, due to high PFDA molar concentrations (89%) and a smoother surface. Unsurprisingly, there was a slight decrease in WCA with NVP content in the copolymer films (Figure S9), with the lowest WCA of 114° for pPFDA(3)-pVP(3), but all films remained highly hydrophobic. The long chain of −(CF_2_)_8_-F may predominantly contribute to the surface property and shield the effect on the surface energy from the small NVP groups. This retained hydrophobicity in the copolymers is a practical advantage for optical devices. However, we have not characterized the hysteresis between advancing and receding contact angles to assess whether the surface undergoes reconstruction upon exposure to water. Previous studies of PFDA copolymers have shown that crystallinity and cross-linking can hinder this reconstruction, reducing the contact angle hysteresis. On the other hand, amorphous films of perfluorinated acrylates (e.g., iCVD PFDA synthesized under certain conditions, linear PFDA copolymers, and fluoroacrylates with shorter pendant alkyl groups) have been shown to display higher contact angle hysteresis.^56,62,91,96,97^ Given the amorphous film morphology and inclusion of hydrophilic NVP, poly(PFDA-*co*-VP) films may display higher hysteresis than semicrystalline pPFDA, but this will be a subject of future studies.

### 2.4 Optical Properties

**Figure 5:**
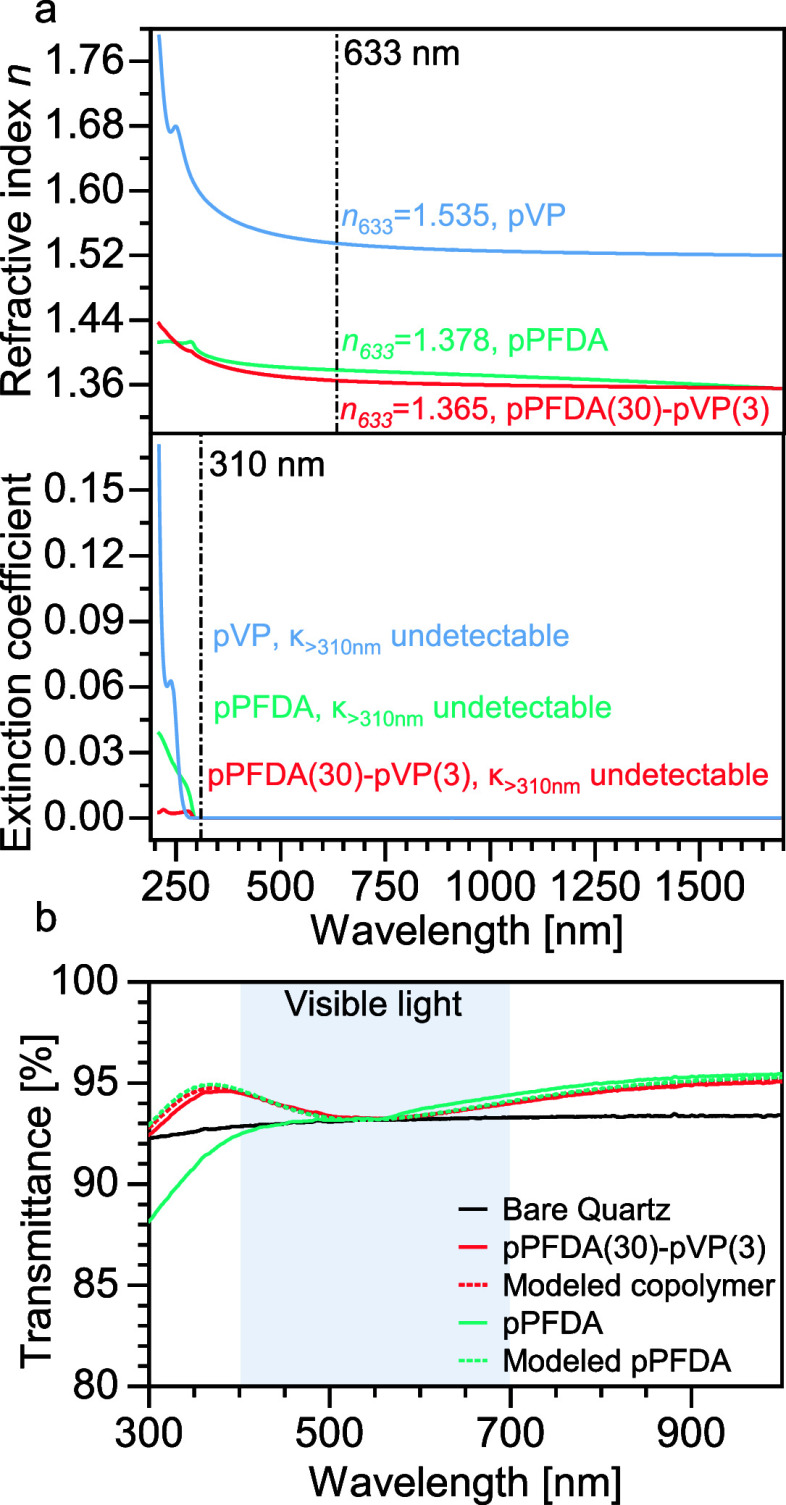
Optical properties. (a) Refractive index and extinction coefficient spectra of pPFDA, pVP, and pPFDA(30)-pVP(3). (b) Measured and modeled transmittance of quartz wafers coated by 200 nm thin films made by pPFDA and pPFDA(30)-pVP(3). The modeled transmittance does not account for scattering loss.

The optical properties of the polymer films were characterized by spectroscopic ellipsometry; the refractive index (*n*) and extinction coefficient (κ) spectra of the films are provided in Figures 5a and S10. Normal dispersion of the refractive index was observed for all films at wavelengths above 300 nm, consistent with dielectric materials. The refractive indices of the homopolymers pVP and pPFDA are 1.535 and 1.378 at 633 nm, respectively. After copolymerization of NVP and PFDA, the copolymers surprisingly showed refractive indexes lower than those of pPFDA despite the incorporation of NVP. pPFDA(30)-pVP(3) with the lowest NVP content (11%) had a refractive index of 1.365 at 633 nm, while pPFDA(3)-pVP(3) with the highest NVP content (29%) had an index of 1.376. The other copolymer compositions, pPFDA(15)-pVP(3) and pPFDA(6)-pVP(3), had almost identical indexes of 1.368. It is interesting to note that regardless of the change in NVP content from 11% to 29%, the refractive indexes of the copolymers vary slightly and are far below the index of pVP. This is highly desirable for the preparation of low-index materials. In fact, the index of copolymer films is lower than the index of conventional MgF_2_ (1.38 at 633 nm). Achieving the low index in the copolymer films is a function of two complementary effects. First, even though the NVP concentration increases to 29 mol % in the copolymer, the high atomic concentration of fluorine, possessing low molar refractivity, is preserved. As the PFDA content in the copolymer decreases from 100% to 71 mol% for pPFDA(3)-pVP(3), the atomic fraction of fluorine in the film decreases from only 53.1% to 48.2% (excluding hydrogen). This is a function of each PFDA monomer contributing a large, perfluorinated side chain (−C_10_H_4_F_17_) while the NVP pendant moiety is much smaller (−C_4_H_6_NO). Second, an additional reduction in the copolymer refractive indexes is due to the suppressed crystallization, which led to a less dense amorphous phase (larger molar volume).

The extinction coefficient of a material is the imaginary part of its complex refractive index and indicates the attenuation of light in the film. An extinction coefficient less than 10^–6^ over the visible wavelengths is typically required for optical components to be classified as “highly transparent”. The extinction coefficient of the materials was measured by the same ellipsometric model for refractive index and is shown in Figure 5a. Kramers–Kronig’s relation was enforced to preclude unphysical results. All the copolymers as well as the homo-pPFDA and homo-pVP had extinction coefficients less than 1 × 10^–6^ from 310 to 1690 nm. The excellent nonabsorbing property of the homopolymers was successfully imparted to the copolymers and resulted in a very broad highly transparent window from UV to near-IR wavelengths.^99^

The transparency of the coating was further assessed through the characterization of the transmittance of 200-nm-thick polymer coatings deposited on quartz plates. The ideal, expected transmittance of quartz coated with pPFDA homopolymer and pPFDA(30)-pVP(3) was modeled based on the optical constants and film thicknesses and compared with their measured values in Figure 5b. The calculated transmittance of the coated quartz showed higher transmittance than the uncoated quartz over the entire spectrum, a result of antireflection since both polymers have indexes lower than that of quartz (*n*_633_ = 1.459, *n*–*k* plotted in Figure S10). The coating structures were not optimized for antireflection in the visible range for this analysis. The measured transmittance of quartz coated with either pPFDA or pPFDA(30)-pVP(3) closely overlapped with the calculated value in the near-infrared region. However, at wavelengths smaller than approximately 530 nm, the transmittances of the homopolymer and the copolymer films deviated, with the transmittance of pPFDA monotonically decreasing at lower wavelengths. This transmittance loss is a result of scattering by the crystallites of pPFDA at wavelength-comparable length scales, which was not included in the idealized optical model. In contrast, the experimentally measured transmittance of the copolymer films with suppressed crystallization perfectly matched the theoretical optical model from 1000 to 300 nm. This further confirmed the necessity and success of our effort to suppress the crystallization of pPFDA for application as low-index optical coatings. The transmittance of quartz coated with pVP and copolymers at other compositions is provided in Figure S11. All copolymers showed similar transmittance spectra higher than that of bare quartz and did not have any obvious scattering loss at lower wavelengths. The transmittance was reduced with the pVP homopolymer coating, despite the absence of haze, due to increased reflection losses as a consequence of its refractive index being higher than that of quartz.

The design rules for selecting candidate comonomers for copolymerization with PFDA to yield amorphous bulk and smooth surface morphologies, enhanced thermal stabilities, and reduced refractive indexes as achieved with poly(PFDA-*co*-VP) have not been fully established, but general guiding principles can be postulated. To maximize the disruption of crystallization, the comonomer should insert in an isolated fashion between PFDA units in the copolymer backbone; long segments of repeated comonomer should be minimized, as the effect of each comonomer residue is reduced in this scenario and offers the potential for microphase separation. Based on the *Q*–*e* scheme, the comonomer should have an *e* value (double bond polarity) that is opposite to that of PFDA to favor the monomer crossover propagation reactions during copolymerization. Simultaneously, the comonomer’s *Q* parameter, which represents comonomer reactivity, should be much lower than that of PFDA’s so that homopropagation of the comonomer is kinetically unfavored. Notably, *N*-vinylpyrrolidone fulfills this requirement perfectly (*Q* = 0.088, *e* = −1.62).^81^ Moreover, for low-index applications, comonomers should be selected that will preserve or ideally reduce the ratio of molar refractivity to molar volume in the films. Comonomers with relatively small pendant groups (small molecular volume) should be favored, and cross-linking, which increases film density, should be avoided. The comonomers should also be composed of elements with low polarizability (aromatic groups and halogens should be avoided).

Few studies of the linear copolymerization of PFDA with comonomers are available in the literature. Copolymers of methacrylic acid and PFDA show an increased index of refraction (∼1.40) at comparable comonomer concentrations in the film.^100^ The *Q*–*e* values of methacrylic acid are 0.98 and 0.62, respectively, suggesting that the comonomers are more likely to undergo ideal copolymerization.^81,101^ Furthermore, the methacrylic acid groups can dimerize in the bulk film due to cooperative H-bonding interactions, further increasing the film density. 4-vinylpyridine (4VP), with *Q*–*e* values of 2.47 and 0.82, has also been copolymerized with PFDA, again suggesting a more ideal copolymerization behavior.^102,103^ Combined with the high refractivity of the pyridine ring, it is not surprising that the refractive index increases with the 4VP content. However, the extent of crystallinity has not been quantified in either copolymer system, precluding conclusions about the effect of each variable. From widely available commodity vinyl monomers, ethyl vinyl ether (*Q* = 0.018, *e* = −1.8) and vinyl acetate (*Q* = 0.026, *e* = −0.88) are hypothesized as potential alternatives to NVP that can achieve comparable copolymer film properties.^102^

### 2.5 Six-Layer Optical Interference Coating

**Figure 6:**
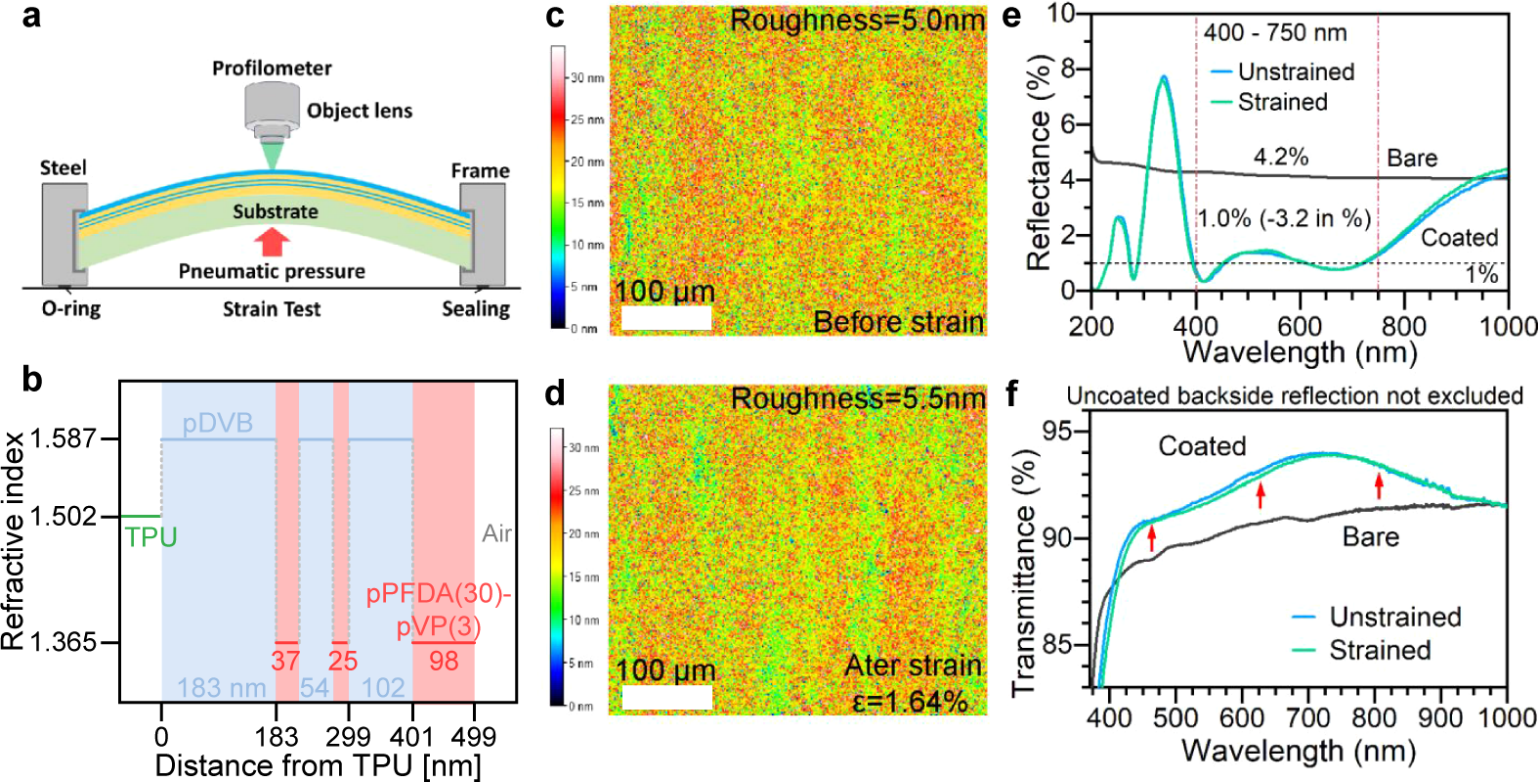
Practical device demonstration and characterization. (a) Illustration of a six-layer polymeric interference coating deposited on a transparent and flexible TPU substrate, including the setup of the strain test coupled with optical profilometry. The TPU substrate is round and fixed within a circular steel ring. (b) Index and thickness profile of the six-layer coating. Surface topography by profilometry of the six-layer coated TPU (c) before and (d) after 1.64% equibiaxial strain demonstrating the film’s structural integrity. The antireflection effect by the six-layer polymeric interference coating is confirmed by (e) reflectance and (f) transmittance measurements with uncoated backside reflection included (contributing to the reflection losses).

To demonstrate the performance of the material in a practical application, a six-layer optical interference coating was designed with a p(PFDA*-co-*VP) copolymer thin film as the low-index layer component. A schematic of the six-layer coating on a thermoplastic polyurethane (TPU) is shown in Figure 6a with an illustration of the strain test where an equibiaxial strain was pneumatically applied while the surface topography of the coating was examined by optical profilometry to assess its mechanical durability. The six-layer structure constitutes an alternating stack between a low and a high refractive index layer. Poly(divinylbenzene) (pDVB) was chosen as the high-index material for its relatively high index of 1.59 (*n*_633_) among polymers and its availability by iCVD.^104^ The thickness of each layer in the stack was optimized for a broadband antireflection effect over the visible wavelength range of 400 to 750 nm. The optical design is detailed in Figure 6b. The six-layer coating was fabricated by iCVD through the sequential growth of each material on the TPU substrate. An in situ ellipsometer was incorporated into iCVD so the film thickness of each layer was individually controlled according to the optical design. The ellipsometry data of the sequential depositions are presented in Figure S12.

Here, to assess the mechanical performance of the six-layer polymeric coating, we employed a method that characterizes the surface topography evolution before and after an aggressive equibiaxial strain of 1.64% by combining optical profilometry and a pneumatic strain device. Such a method was previously used to study the mechanical flexibility and durability of a barrier layer, a two-layer polymeric antireflection coating, and several inorganic coatings in our previous work.^7,60^ The strain of 1.64% was the upper limit of our equipment. Figure 6c shows the topography of the six-layer coated surface. No features of mechanical defects such as cracks, wrinkles, buckles, blisters, pinholes, bubbles, or delamination were observed over an imaged area of 400 × 300 μm. Instead, the surface was very smooth with a roughness (root-mean-square height) of 5.0 nm, indicating great film quality consistent with the SEM results even when six layers of polymers were stacked. After undergoing mechanical strain, the surface was imaged again and shown in Figure 6d. No meaningful change was observed compared with that before the strain except for an insignificant increase in roughness to 5.5 nm. No cracks or any mechanical defects detectable by the profilometry were generated during the strain. The six-layer polymer coating withstood the equibiaxial strain and remained intact, which proved not only that the p(PFDA-*co*-VP) copolymer proposed in this work was mechanically flexible and compliant upon strain but also that such properties were preserved in and extended to a coating of six layers. Such performance would enable flexible optical coatings to be engineered with high complexity while maintaining durability. Figure 6e,f displays the reflectance and transmittance of the six-layer coated and uncoated TPU compared before and after the strain. An excellent broadband antireflection effect was observed—the front reflectance of TPU reduced from 4.2% (uncoated) to 1% after coating over the entire visible range from 400 to 750 nm. The corresponding improvement in transmittance over a broadband of light was also observed for the coated sample. Neither reflectance nor transmittance showed any meaningful change upon strain for the coated sample—the copolymer and the six-layer coating preserved their optical performance upon the mechanical deformation.

## 3 Conclusion

New low refractive index materials of p(PFDA*-co-*VP) were readily fabricated for the first time using the solvent-free, one-step iCVD technique. The NVP comonomer was found to suppress the crystallization of the long-chain polyfluoroacrylates pPFDA, giving it excellent transparency while preserving its desirable low refractive index. The refractive index of this copolymer is only 1.365 at 633 nm, and unlike homopolymer pPFDA films, it showed no detectable haze or absorption from 310 to 1690 nm. The copolymerization also exhibited fast kinetics that delivered a high deposition rate greater than 140 nm min^–1^. Furthermore, p(PFDA*-co-*VP) films were shown to be exceptionally smooth (RMS roughness <1 nm), hydrophobic (WCA ∼ 121°), uniform (thickness ±1.5% over 2 × 2 cm), and thermally stable (0.4% thickness loss at 200 °C for 1 h). To demonstrate the practical optical application of this material, a six-layer interference coating consisting of alternating low-index p(PFDA*-co-*VP) (*n*_633_ = 1.36) and high-index poly(divinylbenzene) (pDVB) (*n*_633_ = 1.59) was fabricated on an elastomeric optical element. A completely polymeric six-layer interference coating with precise independent thickness control of each individual layer with nanometer-level precision was demonstrated for the first time by iCVD. A reflectance of only 1% was achieved, and the six-layer coating withstood aggressive biaxial strain without fracturing or other compromises in optical performance. In summary, the p(PFDA*-co-*VP) copolymers described in this report possess tremendous potential for practical application as flexible low-index optical coating materials due to their excellent transparency, surface uniformity, smoothness, hydrophobicity, and thermal stability as well as tunability by iCVD.

## 4 Experimental Section

### 4.1 Film Synthesis

The polymer thin films were synthesized in situ on substrates in a custom-built initiated chemical vapor deposition (iCVD) system. The iCVD consists of a 5.5 in. sided hexagonal vacuum chamber with an in situ ellipsometer employed to monitor and control the film thicknesses. An array (6.5 × 6.0 in.) of resistively heated filaments (Master Wire, Nichrome 80) was fixed 1 in. above the substrate. The filament was powered by a DC supply (TDK Lambda, GEN 150-5-USB-U). To precisely control and maintain the substrate temperature, Si and quartz wafers were placed on an aluminum temperature stage (5.8 × 6.4 × 0.5 in.) (Wieland MicroCool) liquid-chilled by a cooling/heating recirculation system (VWR, AD07R-20). The pressure in the reaction chamber was controlled by a digital pressure controller (MKS 600 Series) connected to a downstream throttle valve (MKS 653B) and pressure gauge (Brooks, XacTorr CMX100). All detailed deposition parameters can be found in Table S1.

1*H*,1*H*,2*H*,2*H*-Perfluorodecyl acrylate (PFDA, Oakwood, 98.9% GC purity), *N*-vinylpyrrolidone (NVP, Sigma-Aldrich, 99.7% GC purity), and divinylbenzene (DVB, Alfa Aesar, mixture of isomers) were kept in glass monomer jars in a water bath with a fixed temperature of 25 °C. Argon as a carrier gas was sparged (Airgas, high purity grade) through the monomers by a bubbler to deliver monomer vapor into the chamber. Mass flow controllers (MFCs by MKS) were utilized to regulate the flow rates of carrier gas for PFDA, NVP, and DVB. Di-*tert*-butyl peroxide (TBPO, Acros Organics, 99% purity) was used as the initiator, and its vapor was delivered without carrier gas and maintained at a constant 2.0 sccm by an MFC (Horiba STEC, SEC-4400). Flow rates of carrier gas varied as designed in each experiment. The polymers are labeled with the carrier gas flow rate of each monomer. For example, pPFDA(30)-pVP(3) indicates that carrier gases of NVP and PFDA monomer are 3 and 30 sccm, respectively.

Si (100 mm diameter) and quartz (2 in. diameter) wafers were purchased from University Wafer. Aliphatic thermoplastic polyurethane (TPU) membranes were provided by Sheedom Co. The TPU was stretched and fixed to a stainless-steel ring frame by using a Schmidt ring press. An O-ring was installed on the backside of the frame to provide gas sealing, so the TPU membrane could be pneumatically deformed. The thickness of the TPU membrane was measured to be about 230 μm by a micrometer.

### 4.2 Material Characterization

Fourier transform infrared spectroscopy (FTIR) was conducted using a Thermo Fisher Scientific Nicolet iS50. The spectra of all polymers were collected in transmission mode from a film deposited on a Si wafer. The film thickness at the imaging spot was precisely measured by using an ellipsometer to quantitatively normalize the absorbance. The films were subsequently scratched off to take the spectra of the Si substrate, which were then subtracted from the sample spectra. All of the intensities of the curves are quantitatively comparable. For all measurements, the resolution was set to 4 cm^–1^ and a total of 64 scans were integrated to improve the signal-to-noise ratio of the spectra.

X-ray photoelectron spectroscopy (XPS, Kratos Axis Ultra DLD) was carried out to quantitatively determine the copolymer compositions. A monochromatic Al-Kα source was employed with an anode voltage of 15 kV and an emission current of 10 mA. A 160 eV passing energy and a 1 eV step were used for survey scans from 1200 to −5 eV. A 20 eV passing energy was used for high-resolution scans for the analysis of individual elements. Three scans were conducted for each high-resolution spectrum while one scan was used for each survey to minimize the decomposition of fluoropolymers under the prolonged X-ray exposure.^105^ Molar concentrations of PFDA and NVP in copolymers were determined by the atomic concentration ratio of fluorine/nitrogen acquired from survey scans and used as the nominal concentrations of all copolymer species. All XPS peaks were referenced to C 1s of C–C defined at 284.8 eV. Peak deconvolution was conducted via CasaXPS (version 2.3.25) software.

The complex refractive index (*n* and κ) and roughness of all polymers were modeled based on ellipsometry (J.A. Woollam, RC2) data of a 100 nm thin film (400 nm for pVP, 175 nm for pDVB) coated on a Si wafer and collected at three different incident angles (50°, 60°, 70°); the multiangle experiments were conducted to improve the reliability and accuracy of the results. GenOsc models (Kramers–Kronig relation embedded) were used to fit the wavelengths between 210 and 1690 nm. The optical constants of uncoated TPU and quartz used in the model were also obtained separately. During the deposition, the film was monitored by an in situ ellipsometer (J.A. Woollam, iSE). The in situ ellipsometric data of wavelengths from 400 to 1000 nm were collected and fitted with absorption-free Cauchy models to acquire thickness. For the in situ monitoring of the coating growth on quartz and the multilayer on TPU, the optical constants of the growing films were not fitted; instead, they were precollected by the multiangled RC2 and used as input. The transmittance of quartz and TPU samples was measured by the UV–vis (Thermo Fisher Scientific Evolution 300). For transmittance, different polymers were deposited on a 2 in. quartz wafer at 200 nm thickness. The reflectance of TPU samples was characterized by a normal-angle reflectometer (Filmetrics, F-20 UV). Both the coated and uncoated TPUs were backside-taped with white tape at a similar refractive index to diffuse the backside reflection so only front-side reflectance was collected and compared. The six-layer antireflection coating was designed by simultaneously fitting the thickness of each layer to minimize the reflectance over the 400–750 nm visible range using CompleteEASE (6.64d) software. The simulated transmittance was also generated by CompleteEASE.

Differential scanning calorimetry (DSC, TA Instruments Q2000) was used to investigate the crystallization behavior of the polymers. For every material, approximately 2 mg of material was scratched from a coated Si wafer as-deposited and loaded into a hermetically sealed DSC pan. The sample was then heated from 30 to 120 °C under a N_2_ purge at 5 °C·min^–1^.

A thermal cell (J.A. Woollam, Linkam THMS600) was used with the ellipsometer for in situ monitoring of the thickness evolution of the prepared thin films as a function of temperature. For the staircase ramp test protocols, a coated Si wafer was placed in the thermal cell and heated at 20 °C·min^–1^ to 100 °C under a nitrogen purge. After being isothermal for 1 h, the temperature would ramp and hold for 1 h again, stepwise at 25 °C intervals until reaching 300 °C, and then cool down to 25 °C at the same rate. For the prolonged isothermal test, the sample in the thermal cell was directly heated to 200 °C at 20 °C·min^–1^ and held for 48 h.

Scanning electron microscopy (Zeiss, AURIGA) was conducted to characterize the surface morphology of polymer thin films coated on Si wafers at an accelerating voltage of 5 kV. A 2-nm-thick Au coating was applied via DC sputtering on the surface to mitigate charge accumulation. A polarized optical microscope (Leica Type 020-520.714) in reflection mode was also used to image the morphology of polymer coatings on Si wafers. Static water contact angles (WCA) of polymer coatings on quartz were collected using a contact angle goniometer (Biolin Scientific, ThetaLite100). The angle was recorded immediately (within 2 s) after applying the liquid droplet onto the surface.

X-ray diffraction spectroscopy (Rigaku XtaLAB Synergy-S, equipped with a HyPix-6000HE HPC detector) was used to characterize the crystalline structure of copolymers and homopolymers with Cu-Kα radiation at room temperature, 50 kV, and 1 mA. Polymer coatings were scraped off from Si wafers using a razor blade and affixed to a nylon loop (0.1 mm ID) with a light coating of viscous oil for powder XRD.

The method of using the equibiaxial strain test of the coated TPU to assess the mechanical flexibility and durability of the polymeric coatings was reported in detail in our previous papers.^7,60^ For the convenience of readers, it is also included in the Supporting Information. The surface topography of the six-layer coating was imaged by a white light interferometer/profilometer (Filmetrics, Profilm3D).

## Supporting Information Available

iCVD deposition settings, copolymerization kinetics calculation, strain test protocol, ellipsometric fittings of the multilayer fabrication, XRD spectra, POM images, as well as additional DSC results, SEM images, contact angle measurement, transmittance, and optical constants (PDF)
