# Supplementary material for: Control of Fluoropolymer Crystallinity for Flexible, Transparent Optical Thin Films with Low Refractive Indexes
Source: Macromolecules. 2025 Jan 29;58(3):1265–78. doi: 10.1021/acs.macromol.4c02242 (PMC11823608; doi:10.1021/acs.macromol.4c02242)
Supplement: Supplementary file 1 — ma4c02242_si_001.pdf [file ma4c02242_si_001.pdf]

Control of Fluoropolymer Crystallinity for Flexible, Transparent Optical Thin Films with Low Refractive Indexes

Yineng Zhao [a†], Fei Hu [b†], Wyatt E. Tenhaeff [a,b,*]

[a] Materials Science Program, University of Rochester, Rochester, New York 14627, USA.

[b] Department of Chemical Engineering, University of Rochester, Rochester, New York 14627, USA.

[†]Yineng Zhao and Fei Hu contributed equally.

*Correspondence E-mail: wyatt.tenhaeff@rochester.edu

Copolymerization kinetics calculation

$$r_1 = \frac{k_{11}}{k_{12}} = \frac{Q_1 e^{-e_1}}{Q_2 e^{-e_2}}; \qquad r_2 = \frac{k_{22}}{k_{21}} = \frac{Q_2 e^{-e_2}}{Q_1 e^{-e_1}}$$

r_1 is the reactivity ratio of addition of PFDA over NVP to a PFDA* radical at a chain end.
r_2 is the reactivity ratio of addition of NVP over PFDA to an NVP* radical at a chain end.
k_{11} is the reaction rate constant of addition of a PFDA to a PFDA radical (PFDA*) at a chain end.
k_{12} is the reaction rate constant of addition of a NVP to a PFDA radical (PFDA*) at a chain end.
k_{22} is the reaction rate constant of addition of a NVP to an NVP radical (NVP*) at a chain end.
k_{21} is the reaction rate constant of addition of a PFDA to an NVP radical (NVP*) at a chain end.

Q_1, Q_2 are measures of reactivity of PFDA and NVP monomers via resonance stabilization; e_1, e_2 are the measures of polarity of PFDA and NVP (molecule or radical) via the effect of functional groups on vinyl groups.

Q_1 and e_1 of PFDA is not directly available. Hence, Q and e of 1H,1H-heptafluorobutyl acrylate (HFBA) are taken as an approximation and used as the Q_1 and e_1 of the PFDA in this calculation. Q_1 and e_1 of 1H,1H-heptafluorobutyl acrylate are 0.96 and +1.34, respectively. Q_2 and e_2 of N-vinylpyrrolidone (NVP) are 0.088 and -1.62, respectively. Note, the calculation of reactivity ratio by Q-e scheme should always be considered an estimation.

Hereby calculated,

$r_1 = 0.21; \qquad r_2 = 0.00076$

Deposition conditions

Table S1. Deposition Parameters

Substrate	Sample	p [mTorr]	I [A]	T_S [°C]	$F_{C\text{-}PFDA}$ [sccm]	$F_{C\text{-}NVP}$ [sccm]	$\bar{d}$[nm]	Objective
Si	pPFDA	200	0.45	35	30	0	377 (100) [a]	
Si	pPFDA(30)-pVP(3)	200	0.45	35	30	3	320 (100)	IR; XPS; DSC; POM; SEM; (n-k; in-situ thermal); kinetics
Si	pPFDA(15)-pVP(3)	200	0.45	35	15	3	390 (102)	
Si	pPFDA(6)-pVP(3)	200	0.45	35	6	3	294 (102)	
Si	pPFDA(3)-pVP(3)	200	0.45	35	3	3	385 (104)	
Si	pVP	750	0.8	20	0	3	402	
Si	pVP [b]	200	0.45	35	0	3	-	Kinetics
Si	pPFDA	200	0.7	25	30	0	-	
Si	pPFDA	750	0.7	25	30	0	-	
Si	pPFDA	1000	0.7	25	30	0	-	
Si	pVP	200	0.7	25	0	3	-	
Si	pVP	750	0.7	25	0	3	-	Kinetics [c]
Si	pVP	1000	0.7	25	0	3	-	
Si	pPFDA(30)-pVP(3)	200	0.7	25	30	3	-	
Si	pPFDA(30)-pVP(3)	750	0.7	25	30	3	-	
Si	pPFDA(30)-pVP(3)	1000	0.7	25	30	3	-	
Quartz	pPFDA [d]	750 (200)	0.45	35	30	0	204 (34)	
Quartz	pPFDA(30)-pVP(3)	200	0.45	35	30	3	200	
Quartz	pPFDA(15)-pVP(3)	200	0.45	35	15	3	207	Transmittance; contact angle
Quartz	pPFDA(6)-pVP(3)	200	0.45	35	6	3	211	
Quartz	pPFDA(3)-pVP(3)	200	0.45	35	3	3	200	
Quartz	pVP	750	0.8	20	0	3	206	
TPU	pPFDA(30)-pVP(3)	200	0.45	35	30	3	ref. to Fig. 6b	Six-layer interference coating
TPU	pDVB	500	0.7	35	$F_{C\text{-}DVB}$ = 5 sccm			

$T_{monomer\ jar}$=25°C; Flow rate of initiator (TBPO) vapor: 2.0 sccm; $F_{C\text{-}monomer}$ indicates carrier gas flow rate. Filament power: 15.3 V × 0.45 A = 6.9 W; 24.0 V × 0.7A = 16.8 W; 27.5 V × 0.8 A = 22 W

a. Two thicknesses were deposited for these conditions. FTIR, POM, SEM, XPS, and DSC were measured from the thick films. 100-nm films were used for n-k measurement and the in-situ thermal stability tests by the ellipsometer. Deposition rates from all films were used to study the kinetics.

b. At this condition, pVP deposition rate was extremely slow (0.03 nm min^{-1}); creating a film thick enough for characterization was impractical. Therefore, another condition was used to grow the film to 100 nm at 1.4 nm min^{-1}.

c. These films were grown solely to record their deposition rate at those conditions. The thickness was not controlled.

d. pPFDA deposition at 200 mTorr was too slow (0.1 nm min^{-1}) for a 200 nm film. Hence, after spending 5.5 hours to grow the first 34 nm, it was switched to a higher chamber pressure 750 mTorr to grow the film to 204 nm at a deposition rate of 13.8 nm min^{-1}.

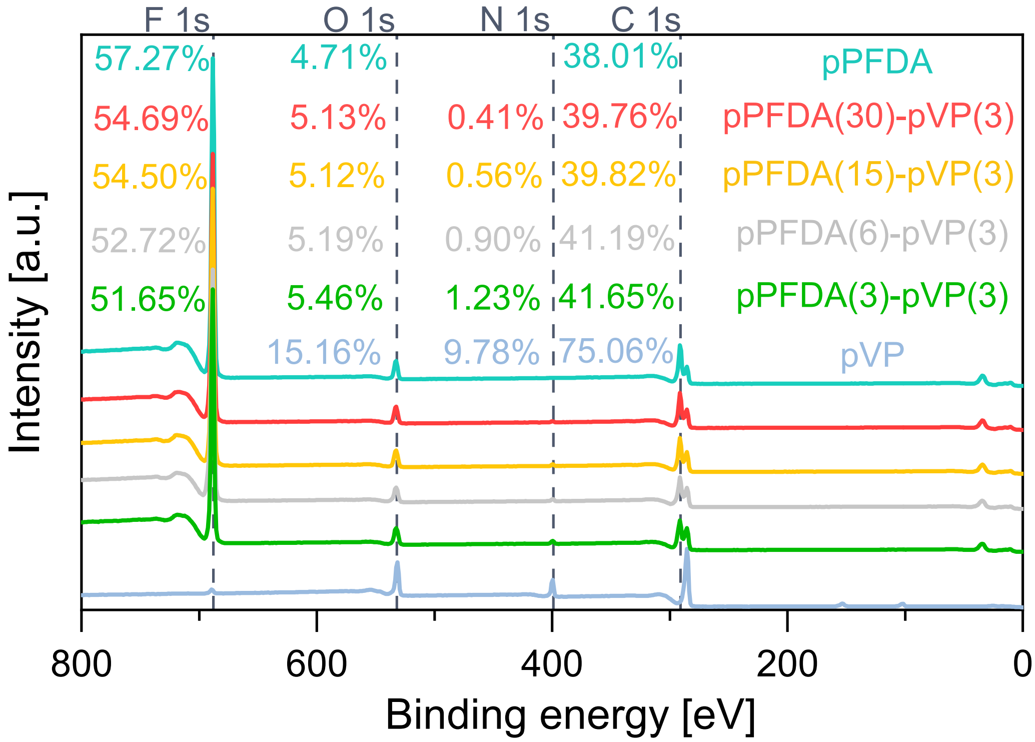

Figure S1. XPS survey spectrum of homopolymer and copolymer films on Si, the percentages in the figure are atomic percentages.

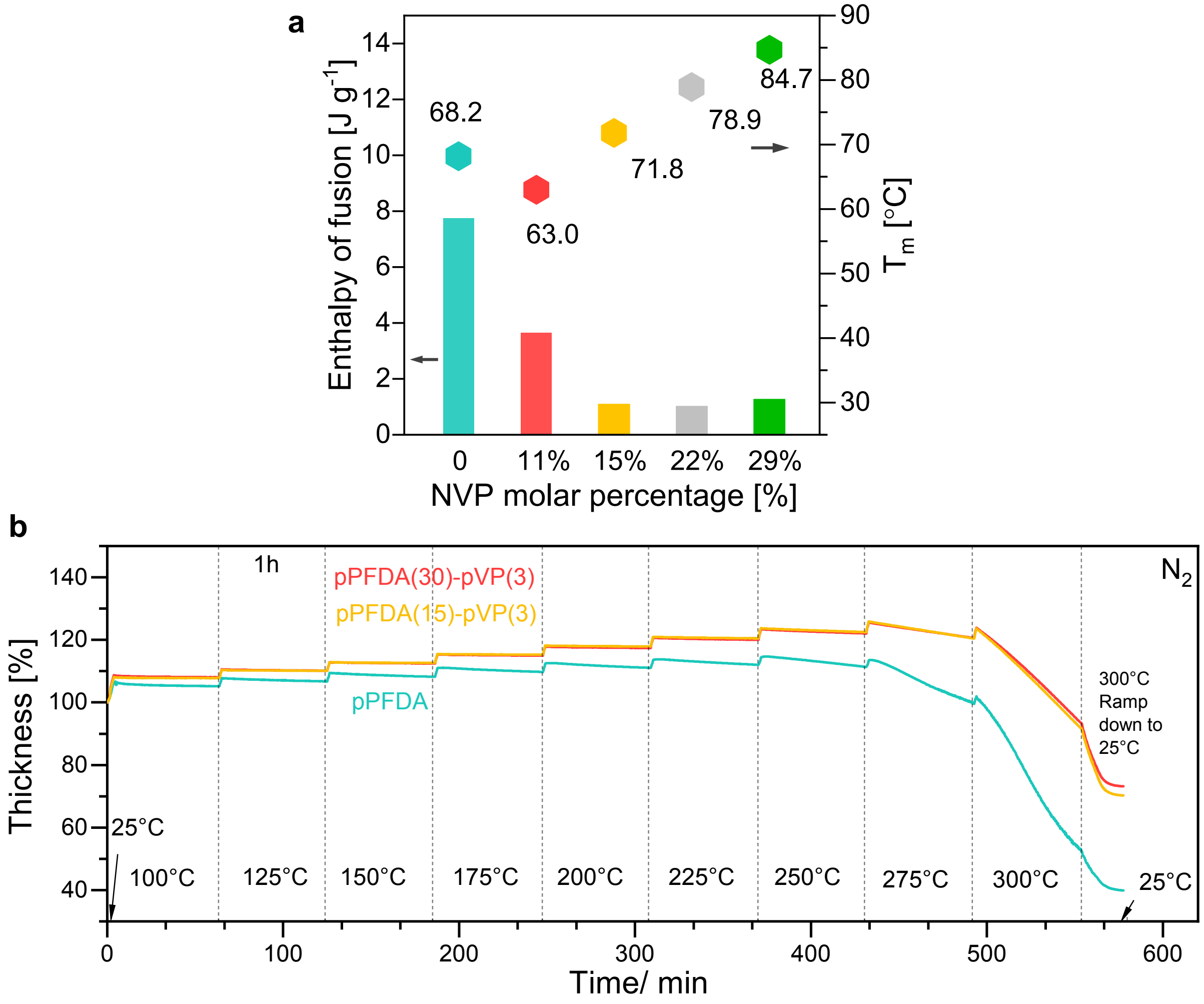

Figure S2. (a) Summary of the DSC results indicating the evolution of enthalpy of melting and melting temperature with copolymer compositions. (b) thickness evolution of polymer thin films pPFDA(15)-pVP(3), pPFDA(30)-pVP(3), and pPFDA at stepwise isothermal conditions monitored by in situ ellipsometer. The ramp rate between set isothermal temperatures is 20°C min^{-1} in a N_2 atmosphere. All samples had nominal film thicknesses of 100 nm and normalized thicknesses were reported in the figures to aid comparison.

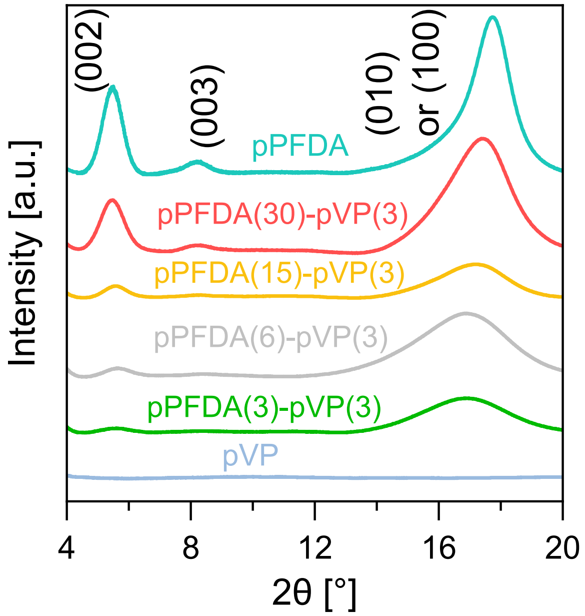

Figure S3. Powder X-ray diffractograms of pPFDA, pVP and copolymer. Baseline is corrected. XRD spectra index reference can be found in Chen et al.[1]

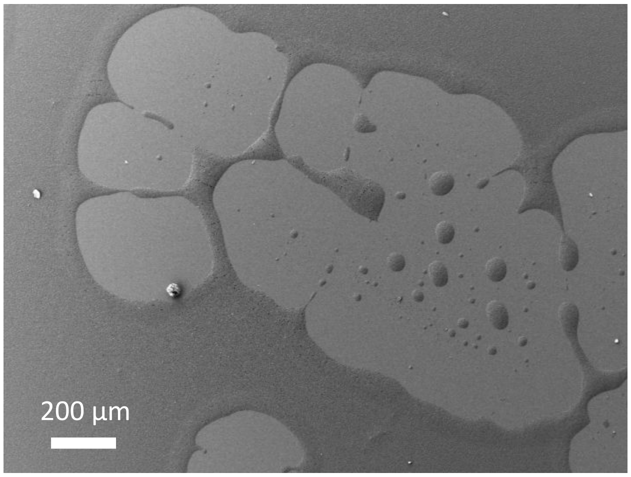

Figure S4. SEM image of pPFDA film dewetting after annealing at 85°C for 2 days.

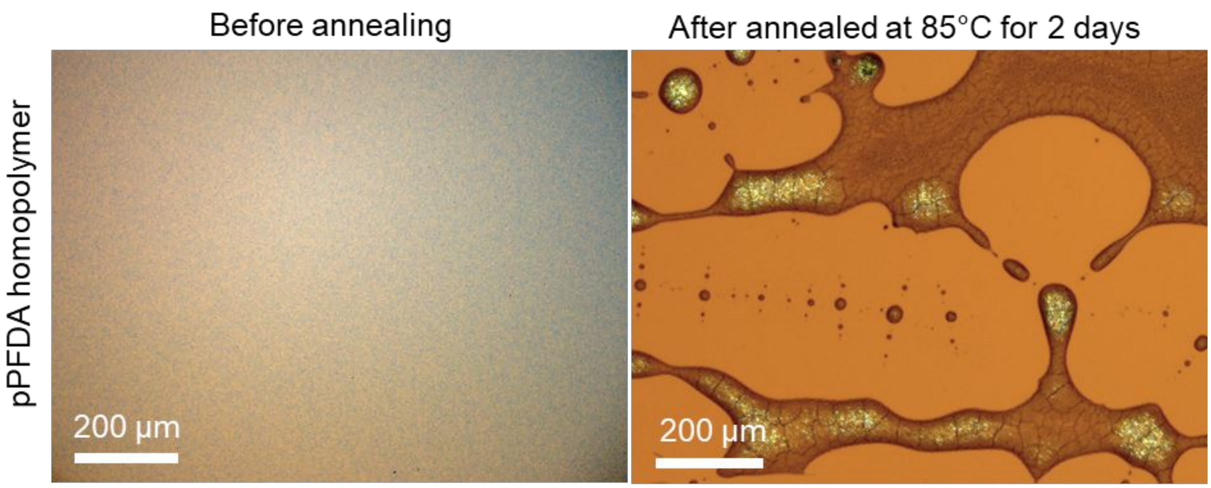

Figure S5. Polarized optical microscope (POM) images of pPFDA film before and after annealing.

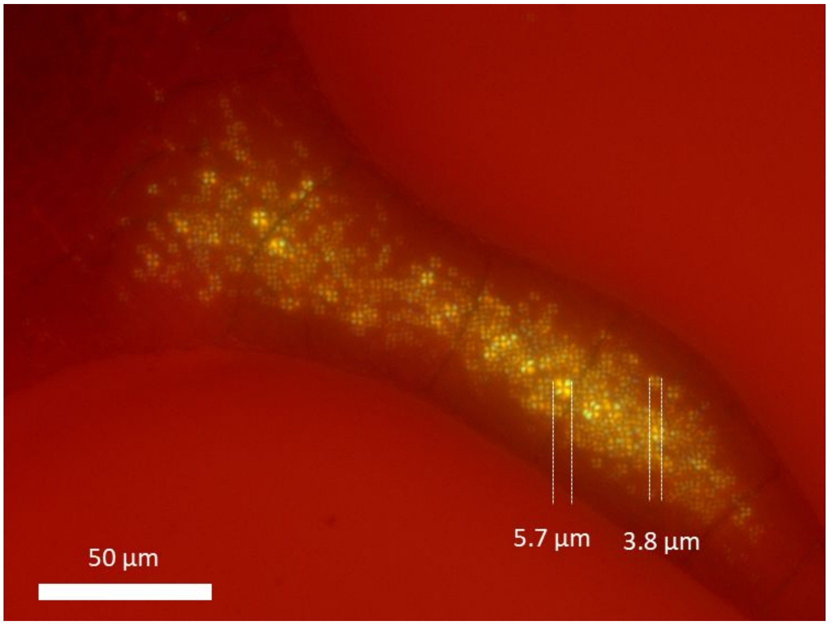

Figure S6. Polarized optical microscope (POM) of annealed (85°C for 2 days) pPFDA film, revealing Maltese cross features.

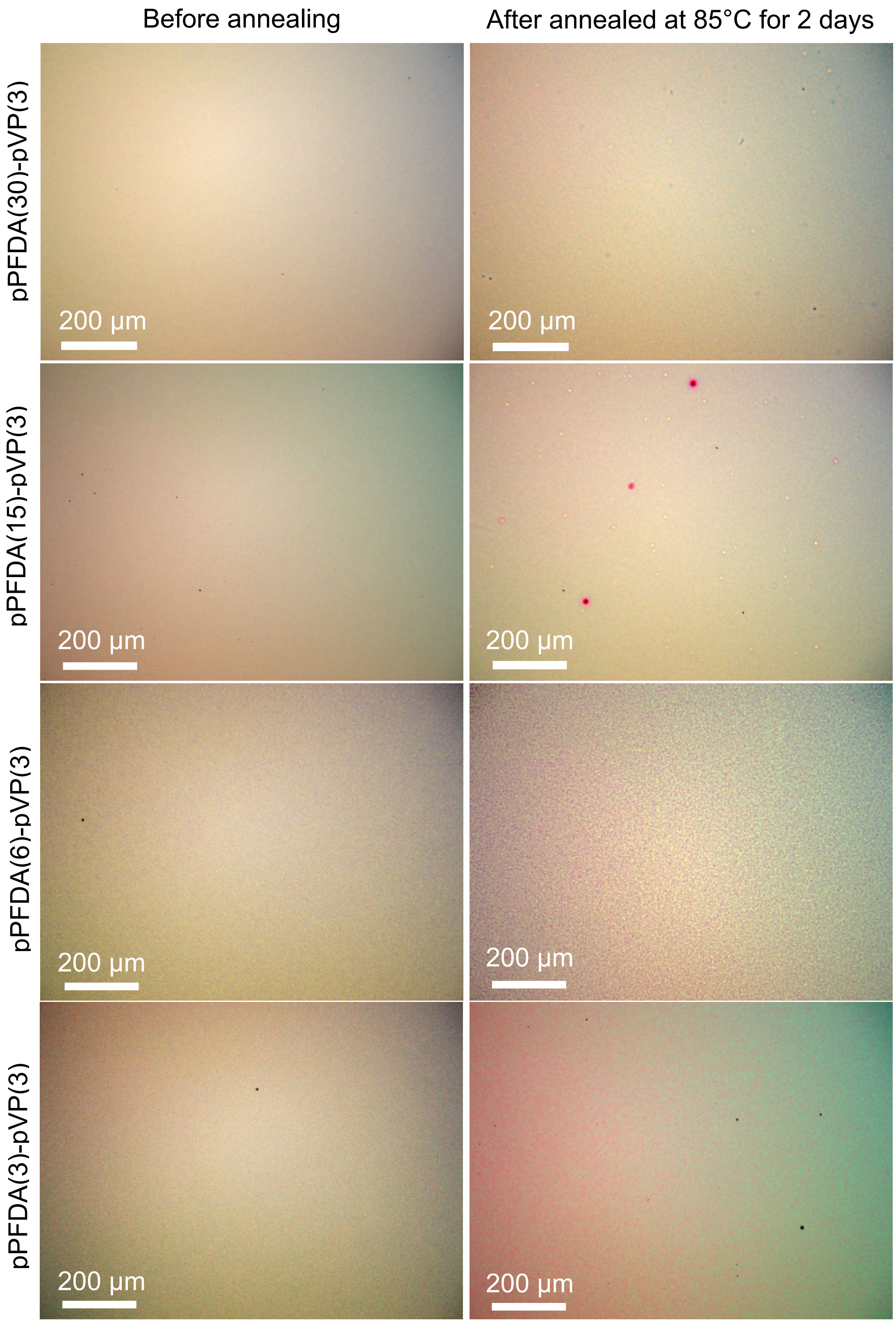

Figure S7. Polarized optical microscope images of copolymer films before and after annealing.

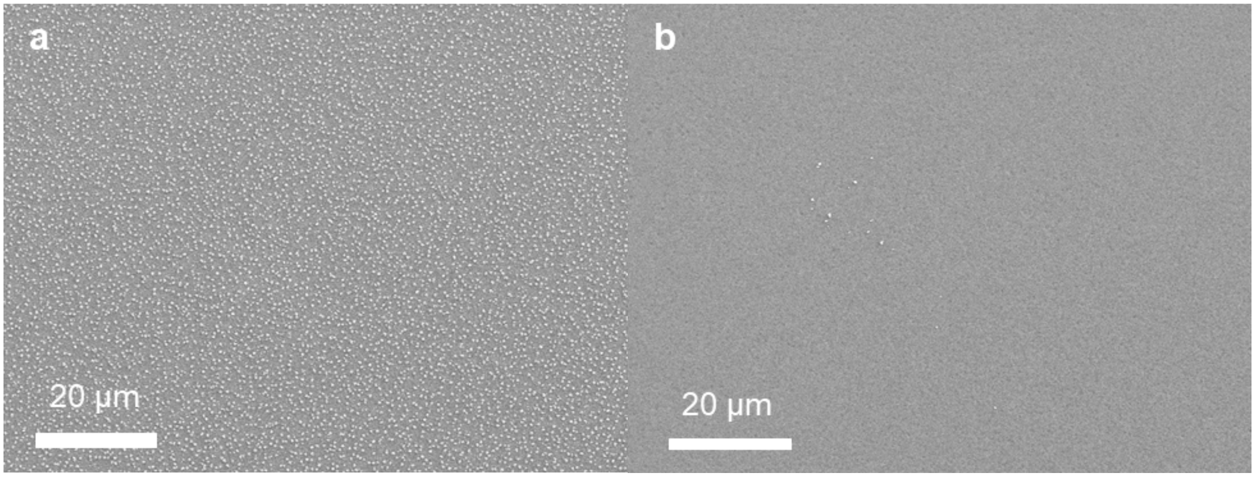

Figure S8. SEM of pPFDA(6)-pVP(3) before (a) and after annealing (b) at 85°C for 2 days.

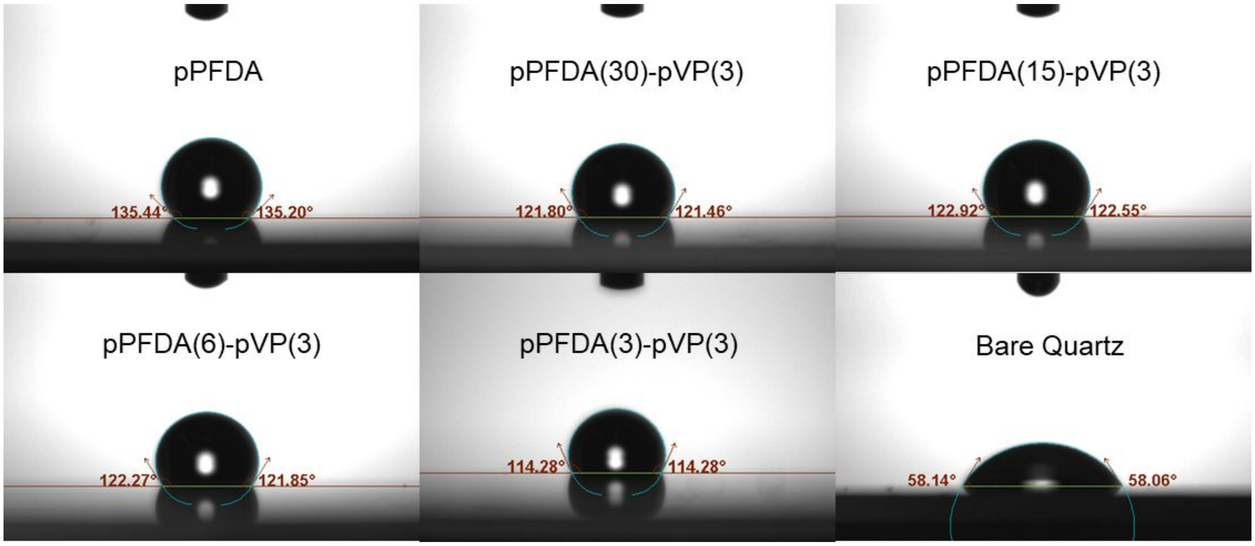

Figure S9. Water contact angles of pPFDA and p(PFDA-*co*-VP) copolymers deposited on quartz wafers.

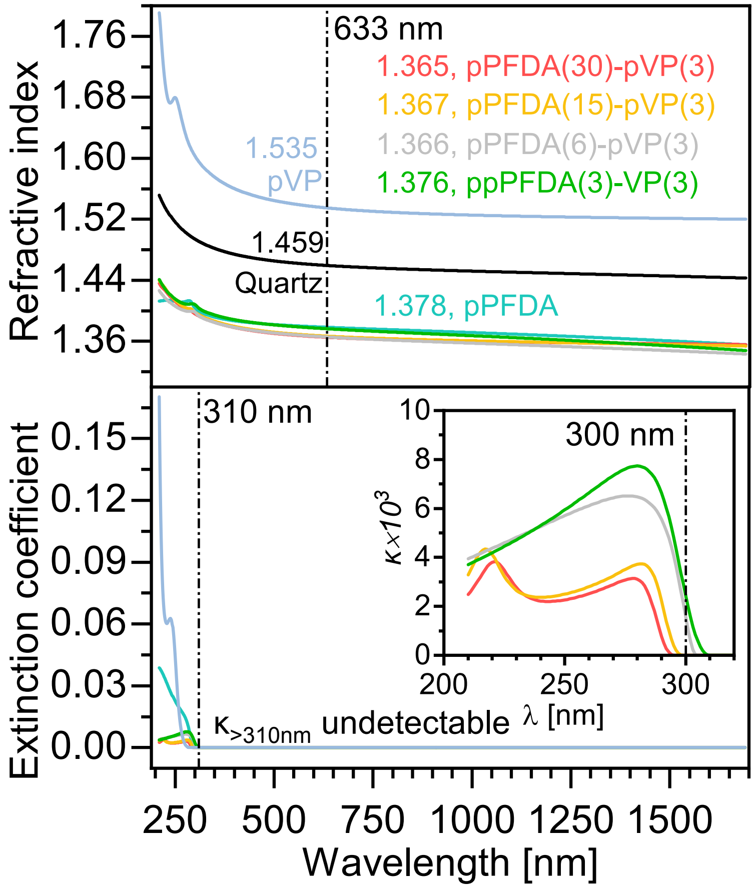

Figure S10. Real refractive indexes (top) and extinction coefficients (bottom) of quartz, pPFDA, pVP, and p(PFDA-*co*-VP) at different compositions. The value displayed are indexes at 633 nm.

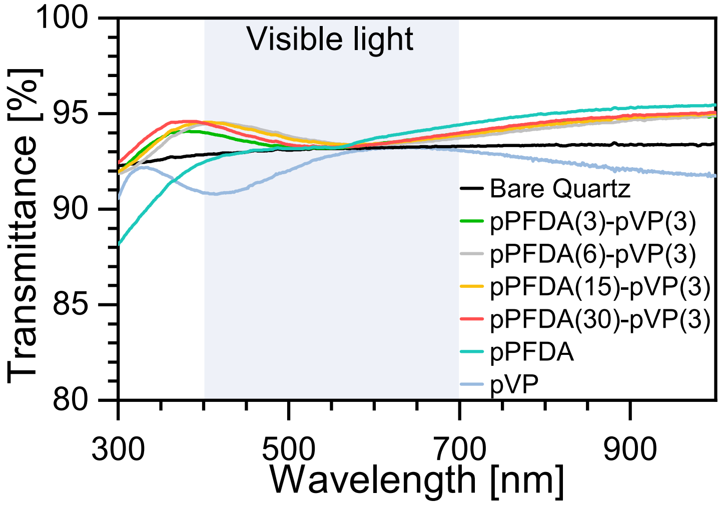

Figure S11. Transmittance of quartz wafers coated by 200 nm of thin films made by pPFDA, pVP, and p(PFDA-*co*-VP) at different compositions.

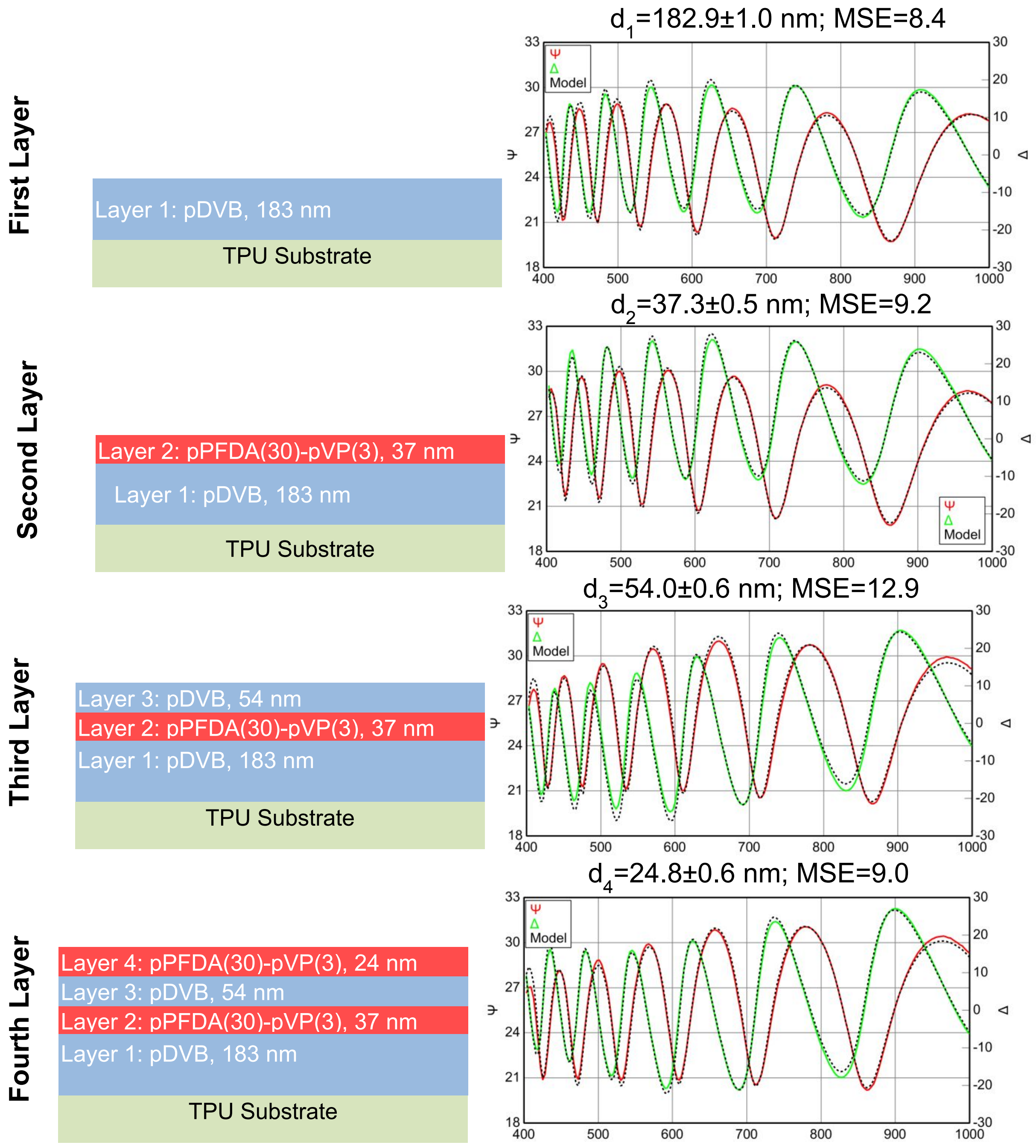

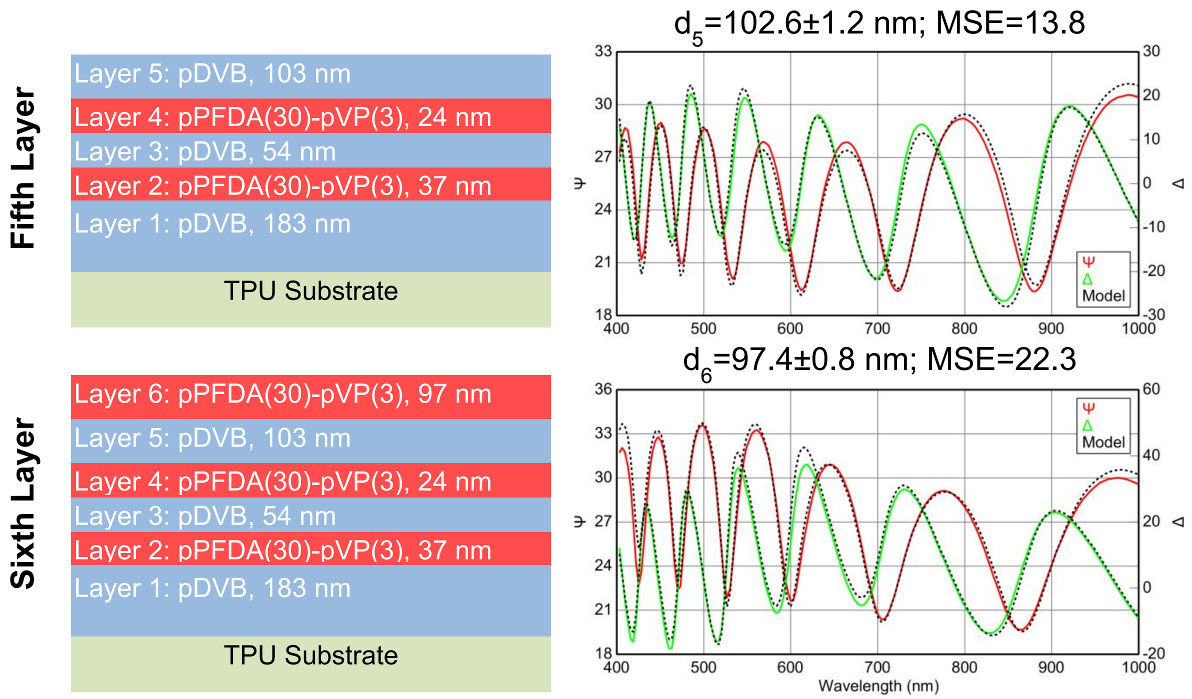

Figure S12. Spectral ellipsometry data after sequential deposition of each layer. The dashed line were the fitted curves using model with the fitted thickness reaching that of the optical design.

Equibiaxial strain test protocol

TPU membranes were fixed on a stainless-steel ring embedded with an O-ring on its bottom. The side with coating was denoted as the front side. Strain was generated by pneumatically pressurize the backside of the membrane by a custom-built 'bulging' device to deflect the membrane. An equibiaxial strain of the pneumatically deformed membrane can be calculated using the following equation:

$$\varepsilon = \frac{\sqrt{A} - \sqrt{A_0}}{\sqrt{A_0}} = \frac{\sqrt{2\pi r h} - \sqrt{\pi a_0^2}}{\sqrt{\pi a_0^2}}$$

where A is the membrane area in the deformed state and A_0 is the membrane area in the undeformed state (A_0= 46.57 cm^2). The a_0 is the radius of the membrane in the undeformed state (a_0= 3.85 cm). Assuming a geometrical relation for a spherical cap, valid for a small deformation, one can calculate A from the radius of curvature, r, and the measured deflection, h. The r can be calculated using h and a_0, as shown in the following equation:

$$r = \frac{a_0^2 + h^2}{2h}$$

The membrane deflection was measured by a laser profiler (Keyence, LJ-X8080). The pressure was measured by a pressure gauge (Dwyer DPGAB-04). The correlation between a pressure and a strain was hence calibrated.

Next, the TPU and the ‘bulging’ device were placed under the objective lens of the Filmetrics profilometer. The strain was then generated pneumatically and determined by the pressure reading. The profilometer was focused on the center of the TPU before, at, or after each targeted strain. The same area of 400×300 μm was imaged after the pressure was stabilized and the vibration was dissipated. Filmetrics Profilm3D was used to process the imaged data. The images were flattened using forth-order fitting and artificial interference patterns caused by persistent vibrations were also removed. No smoothing algorithm was used to preserve the original surface information.

References

[1] Z. Chen, T. H. Nguyen, S. M. Rumrill, K. K. Lau, *Advanced Materials Interfaces* **2022**, 9, 2101961.
